# Musculoskeletal disorders and complaints in professional musicians: a systematic review of prevalence, risk factors, and clinical treatment effects

**DOI:** 10.1007/s00420-019-01467-8

**Published:** 2019-09-03

**Authors:** Gabriele Rotter, Katharina Noeres, Isabel Fernholz, Stefan N. Willich, Alexander Schmidt, Anne Berghöfer

**Affiliations:** 1 Institute for Social Medicine, Epidemiology and Health Economics, Charité - Universitätsmedizin Berlin, Luisenstrasse 57, 10117 Berlin, Germany; 2 Kurt-Singer-Institute for Music Physiology and Musicians’ Health, Hanns Eisler School of Music Berlin and University of the Arts Berlin, Charlottenstrasse 55, 10117 Berlin, Germany; 3 Berlin Center for Musicians’ Medicine, Charité - Universitätsmedizin Berlin, Luisenstrasse 13, 10117 Berlin, Germany; 4 Department of Psychiatry and Psychotherapy, Charité - Universitätsmedizin Berlin, Charitéplatz 1, 10117 Berlin, Germany; 5 Department of Audiology and Phoniatrics, Charité - Universitätsmedizin Berlin, Luisenstrasse 13, 10117 Berlin, Germany

**Keywords:** Musicians’ medicine, Occupational medicine, Playing-related musculoskeletal disorders, Prevalence, Incidence, Systematic review

## Abstract

**Purpose:**

Musicians’ practice and performance routines reportedly lead to musculoskeletal complaints and disorders (MCD) that impact their wellbeing and performance abilities. This systematic review aims to assess the prevalence, risk factors, prevention and effectiveness of treatments for MCD in professional musicians and consider the methodological quality of the included studies.

**Methods:**

A systematic literature search was performed in December 2017 using electronic databases and supplemented by a hand search. Case–control studies, cohort studies, cross-sectional studies, interventional studies and case reports investigating the prevalence, risk factors, prevention or treatment effects of MCD in professional musicians or music students (age ≥ 16 years) were included. Quality assessments of the included studies were performed using an adapted version of the “Study Quality Assessment Tools” from the National Heart, Lung, and Blood Institute.

**Results:**

One case–control study, 6 cohort studies, 62 cross-sectional studies, 12 interventional studies and 28 case reports were included and assessed for methodological quality. The study designs, terminology, and outcomes were heterogeneous, as the analyses mostly did not control for major confounders, and the definition of exposure was often vague. Therefore, evidence that being a professional musician is a risk factor for MCD as well as the causal relationship between these factors remains low despite the fact that a large number of studies have been performed.

**Conclusions:**

Studies with high internal and external validity regarding the prevalence, risk factors and effectiveness of the prevention or treatment of MCD in professional musicians are still missing. Further high-quality observational and interventional studies are required.

**Electronic supplementary material:**

The online version of this article (10.1007/s00420-019-01467-8) contains supplementary material, which is available to authorized users.

## Introduction

To a great extent, professional musicians rely on their physical and mental health to guarantee their vocational and artistic positions, meet high audience demands or succeed in competitive settings. Even minor complaints may impair the precision of motion sequences and musical technique, thus creating a major threat to the artist’s existence.

The discipline of musicians’ medicine dates back to the 18th century (Ramazzini 1705). In the 1920s, Kurt Singer first systematically described symptoms of musicians’ vocational diseases and their treatment (Singer and Lakond 1932; Harman 1993). Currently, musicians’ medicine is dedicated to the prevention, diagnosis and therapy of health problems which may arise or have arisen as a result of making music or which have an effect on making music (Spahn et al. 2011).

In clinical practice, musculoskeletal and mental problems, especially performance anxiety, are very common amongst professional musicians (Fishbein et al. 1988). Up to now, several narrative and systematic reviews exist that provide data on a wide range of playing-related symptoms and diseases, with broadly varying prevalence rates (Harman 1982; Zaza 1998; Milan 1996; Zuskin et al. 2005). The varying prevalence rates may mainly be caused by the lack of a precise definition of playing-related symptoms and diseases as well as a lack of coordinated research.

Reviews of publications including patients with musculoskeletal complaints and disorders (MCD) used different definitions of musculoskeletal disorders or simply referenced “overuse syndrome” (Hoppmann and Patrone 1989; Bejjani et al. 1996). In 1998, Zaza (1998) introduced the term “playing-related musculoskeletal disorders” (PRMD), which aggregated the various musculoskeletal disorders while assuming a common etiological factor. The prevalence of PRMD in musicians was thus deemed to be comparable to vocation-related musculoskeletal disorders in other professions.

The most recent systematic review by Bragge et al. (2006) furthered the use of PRMD as an aggregate term for overuse syndrome, repetitive strain injuries or cumulative trauma disorders. The review reported a prevalence ranging between 26 and 93%. Further systematic reviews added only limited information, as they did not use predefined review protocols or used a narrow search strategy (Wu 2007; Moraes and Antunes 2012). Furthermore, previous reviews did not focus on the methodological quality of the included studies.

Since various musculoskeletal disorders were inconsistently summarized under the term PRMD and the methodological quality of included publications was not assessed in previous reviews, we aimed to perform a comprehensive systematic review including published literature without language restrictions, based on an elaborated study protocol, to assess the prevalence, risk factors, prevention and effectiveness of MCD treatment in professional musicians, including the assessment of the methodological quality of the included studies.

## Methods

The research methods and reporting of this study followed the Preferred Reporting Items for Systematic reviews and Meta-Analyses (PRISMA) guidelines (Liberati et al. 2009; Moher et al. 2009) and the recommendations of the Cochrane Collaboration (Green and Higgins 2011). Prior to conducting the review, a study protocol was prepared by all of the review authors, placing special emphasis on study selection, data extraction and quality assessment.

### Criteria for considering studies for the current review

#### Types of studies

Observational studies (case–control studies, cohort studies, cross-sectional studies), intervention studies (controlled clinical trials and pre–post intervention studies without a control group), case reports and case series reporting clinical interventions published in peer-reviewed journals without language or publication date restrictions were included in this review. Studies published in non-peer-reviewed journals, theses, and gray literature were excluded.

#### Types of participants

Participants in the included studies were male or female professional musicians of all musical genres, including music teachers, instrument teachers and music students in higher education institutions (for example, universities, colleges, conservatories). Studies examining mixed professional populations (amateur, semi-professional/professional) or mixed artistic populations (musicians, actors, dancers) were included only if results were reported for the respective subgroups. When the professional status of the participants was not clearly defined in the publication, a consensus of the reviewing authors was obtained. For studies involving high school music students, participants had to be of at least 16 years of age (defined in this review as “adult”) to be included. Publications on mixed populations (children, adolescents, adults) were included only if results were reported for the respective subgroups. The included studies investigated MCD that was potentially caused by or thought to be related to practicing or performing music. This also included dental and jaw diseases, myofascial pain syndromes, craniomandibular dysfunctions, shoulder belt compression syndromes (like thoracic outlet syndrome and congestion syndrome of the upper thoracic aperture) and percussion hemoglobinuria. Studies lacking relevant information on the different types of participants were excluded.

#### Types of interventions

Studies investigating the effectiveness or efficacy of any type of clinical intervention, including interventions using complementary and integrated medicine in the defined study population, were considered.

#### Types of comparisons

A control group comparison was not required to meet the eligibility criteria.

#### Types of outcome measures

Studies were considered if the primary outcomes included the prevalence, incidence or other information about the prevalence of MCD or risk factors for MCD or clinical treatment effects due to an intervention (preventive, therapeutic or rehabilitative). Studies providing only non-clinical treatment effects or validating methods of measurements were not included.

#### Data sources and searches

The electronic databases MEDLINE and EMBASE via OvidSP and via EbscoHost CINAHL, PsycArticles, PsycInfo and ERIC were searched between January 6th, 2015 and December 7th, 2017, with no limit on the publication date.

Hand searches were performed as follows: the scientific journal *Medical Problems of Performing Artists* (*MPPA, until volume 24, issue 4 December 2009, after in MEDLINE*) between February 13th and 27th, 2015 and actualized on December 7th, 2017 as well as the German journal *Fachzeitschrift Musikphysiologie und Musikermedizin* (*FMM*, years 1994–1999 were excluded, because online access was not available), the official scientific publication of the German Association for Music Physiology and Musicians’ Medicine (DGfMM), between February 28th and March 8th 2015 and actualized on December 7th, 2017. Reference lists of seven identified systematic reviews (Zaza 1998; Bragge et al. 2006; Wu 2007; Baadjou et al. 2016; Jacukowicz 2016; Kok et al. 2016; Vervainioti and Alexopoulos 2015) were searched for further studies. The search strategy followed three guidelines: the study population, investigated MCD and study design. The search strategy is shown in Appendix 1.

### Data collection and analyses

#### Study selection

The titles and abstracts of the identified studies were screened for eligibility by two authors (KN, GR). Studies not meeting the inclusion criteria were excluded. The remaining studies were evaluated for inclusion via full-text reviews by two of the four authors (KN, GR, AS, AB). During the full-text review, a predefined checklist form was completed as reported in “Assessment of study quality and dealing with missing data”. For articles not published in English or German, a translated summary was examined for eligibility criteria. Discrepancies in the study selection between authors were resolved in a consensus conference.

#### Data extraction

Data extraction was performed by two of the four authors (KN, GR, AS, AB) using standardized data extraction forms as reported below. Discrepancies in the data extraction between the reviewing authors were resolved by discussion and consensus.

#### Assessment of study quality and dealing with missing data

Depending on the study design, the quality of studies can be assessed using various checklists or scores. A standard assessment tool is the Study Quality Assessment Tool of the National Heart, Lung, and Blood Institute (NHLBI). It provides a thorough assessment of the quality of studies in all medical disciplines. It can be applied to observational studies such as intervention studies and seemed appropriate to be extended by a scoring system for comparison purposes in this review.

To apply comparable quality assessment tools to all of the included study designs, the reviewing authors developed a modified version of the “Study Quality Assessment Tool” from the National Heart, Lung, and Blood Institute. The “Quality Assessment of Observational Cohort and Cross-Sectional Studies,” “Quality Assessment of Before-After (Pre–Post) Studies With No Control Group” and the “Quality Assessment of Controlled Intervention Studies” (NHLBI) were adapted by adding items to the quality assessment tools about the Critical Appraisal Skills Program (CASP), the “CASP-Checklists” (CASP 2013) and the “Methodology Checklists” of the Scottish Intercollegiate Guidelines Network (SIGN). The instructions for application were predefined and adapted in a consensus by all authors. A scoring system was implemented in which one point was given for each item on the form that was fulfilled by the study and one point was subtracted for each item that was not fulfilled. If an item was not applicable to a study, zero points were given. If a study did not report on the requested item, one point was subtracted for that item. The total score was calculated by adding the individual points without weighting for single items. The total number of possible points varied for each evaluation instrument depending on the type of study. Case–control studies could reach a maximum of 14 points, cohort studies and cross-sectional studies could reach a maximum of 16 points, controlled intervention studies could reach a maximum of 18 points and pre–post studies without a control group could reach a maximum of 15 points (Appendix 2). Total scores were not comparable across different types of study designs. The described modified quality assessment tools were used for the quality assessments of the individual studies by two of the four authors (KN, GR, AS, AB). Disagreements over study quality assessment and scoring were resolved by discussion and consensus. If multiple publications were produced from one study, only one quality assessment was performed based on the publication we considered to be the most comprehensive and with the highest methodological quality.

#### Data synthesis

The extracted information included the (1) authors, (2) date of publication, (3) population(s) studied, (4) number of participants, (5) exposure or interventions relevant to the review questions, (6) randomization status; (7) outcomes and (8) results. If these data were not provided, they were marked as missing. The results of individual studies were reported as frequencies in percentages, effect sizes, mean values with standard deviations or standard errors, significance values, odds ratios and confidence intervals. With the exception of percentages, no calculations were made based on the values provided. If a study did not provide quantitative data in numbers, the narratively described results were used.

## Results

### Overview of selected studies

**Fig. 1:**
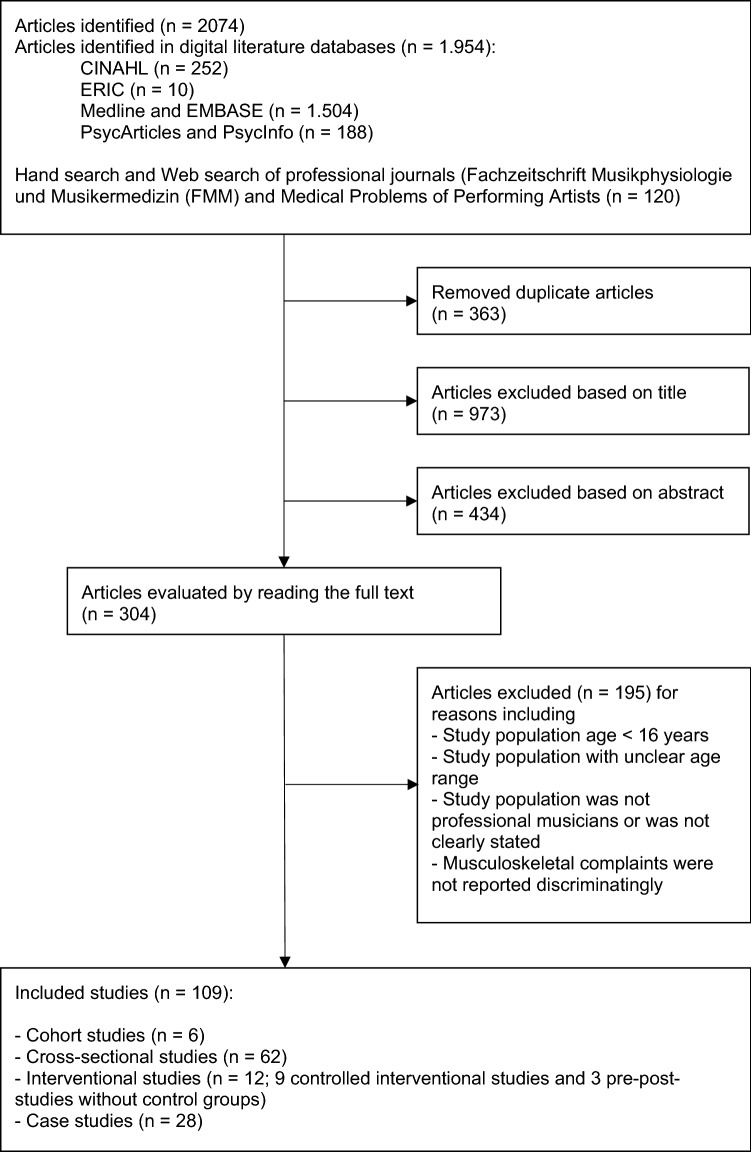
Study selection process

The search strategy identified 2074 articles. Figure 1 depicts the results of the individual steps of the selection process. In total, 109 articles were included in the study, 28 of which were case studies and 81 of which were articles for quality assessment. The 81 articles assessed for methodological quality included 1 case–control study, 6 cohort studies, 62 cross-sectional studies, and 12 interventional studies (9 controlled intervention studies and 3 pre–post studies without control groups).

The characteristics of the included studies are shown in Tables 1 through 6. Across all study types, we found that the different terms used and the definitions of outcome parameters significantly limited the comparability of the included studies. In the “outcomes” columns in Tables 1 through 5, the individual terms used by the studies are cited. Even the abbreviation “PRMD” is used and defined heterogeneously.

**Table 1:** Characteristics and results of the included studies, case–control study

Author (year)	Population	Cases (*n*)	Control (*n*)	Exposure	Outcomes	Results	Quality assessment (*x*/14 points)
Sakai and Shimawaki (2010)	Pianists with overuse disorders and hand problems vs. 62 unaffected pianists in orthopedic consultation of the author, Japan	220	62	Hand size and abduction angle of finger joints	“Overuse”-disorders of the hand	Epicondylitis, muscle pain in the forearm and hypothenar region, De Quervain’s tendinitis and distal tendinitis correlate with variable parameters of small hand size	− 3

The term “PRMD” as “performance-related musculoskeletal disorders” was used in seven publications (Ackermann et al. 2002b, 2011, 2012; Chan et al. 2013; Chan et al. 2014; Khalsa and Cope 2006; Khalsa et al. 2009). Among these, two intervention studies defined performance-related musculoskeletal disorders according to Zaza and Farewell as “any pain, weakness, numbness, tingling or any other symptoms that interfere with your ability to play your instrument at the level you are accustomed to. This definition does not include transient aches or pains” (Ackermann et al. 2002b; Chan et al. 2014; Zaza and Farewell 1997). One study defined the same term as “a musculoskeletal disorder was considered performance-related if the injury occurred during or immediately after playing and the musician specified that playing the instrument was the main contributor to their injury“(Chan et al. 2013). The term “PRMD” as “performance-related musculoskeletal pain disorders” was applied in three other publications (Kenny and Ackermann 2015; Ackermann et al. 2012; Kenny et al. 2016) that used the definition based on Zaza and Farewell (Zaza and Farewell 1997), as mentioned above. The term “PRMD” as “playing-related musculoskeletal disorders” was used in ten studies (Steinmetz et al. 2012; Arnason et al. 2014; Kim et al. 2012; Kaufman-Cohen and Ratzon 2011; Mishra et al. 2013; Kochem and Silva 2017; Monaco et al. 2012; Sousa et al. 2016; de Greef et al. 2003; Rickert et al. 2012). The definitions within the studies were heterogeneous; for example, in one study (Rickert et al. 2012), the term “PRMD” was used without further definition but was used synonymously with “injury” without the specific association that the PRMD was related to playing an instrument. A broad variety of additional terms were applied, sometimes only in one or a few studies (see Tables 1 through 5).

### Case–control study

The included case–control study (Sakai and Shimawaki 2010) investigated the indices of hand and movement angles in 220 pianists in Japan with overuse disorders and 62 unaffected pianists as controls. The authors reported that epicondylitis, muscle pain in the forearm and hypothenar region, De Quervain’s tendinitis and distal tendinitis were correlated with variable parameters of small hand size (Table 1).

#### Quality assessment of the case–control study

The quality of the case–control study (Sakai and Shimawaki 2010) was assessed as a − 3 (out of 14 possible points) due to relevant concerns in the study methodology, such as the absence of a sample size justification and not reporting the details of the study population, inclusion- or exclusion criteria or participant selection (randomly or as a convenience sample). Furthermore, there was no reporting on the blinding of the assessors of exposure/risk factors.

### Cohort studies

Three of the six cohort studies were retrospective evaluations of patient records from a university’s health service, one of which was a follow-up of another study (Manchester and Flieder 1991; Manchester 1988; Manchester and Lustik 1989). The incidence of playing-related disorders of the upper limb and/or hand was reported as 8.5 episodes per 100 music students per year, and 16% had persistent complaints. Risk factors were not evaluated. The fourth cohort study investigated the incidence of work-related musculoskeletal disorders in music teachers and did not identify an elevated arm position of > 30° as a potential risk factor (Fjellman-Wiklund and Sundelin 1998). Another cohort study found that there is generally a moderate degree of disability and pain in violinists, cellists and pianists, though cellists had more disability and pain than the other types of musicians (Piatkowska et al. 2016). The sixth cohort study found that 29% of music students complain about playing-related health issues (mixed somatic and psychological) in their 1st year of study (Nusseck et al. 2017). Details are shown in Table 2.

**Table 2:** Characteristics and results of the included studies, cohort studies

Author (year)	Population	Cases (*n*)	Control (*n*)	Exposure	Outcomes	Results	QA (*x*/14 points)
Fjellman-Wiklund and Sundelin (1998)	Music teachers, Sweden	61	–	Working as a music teacher (plus arm position)	“WMSD’s” (work-related musculoskeletal disorders) and discomfort, modified version of Nordic Questionnaire	Initial 12-month prevalence 80%, after 8 years at 92%, complaints mostly in shoulders, neck and lower back; cumulative “incidences” 0.45 for shoulder, 0.33 for lower back, 0.32 for neck; correlation between frequency of arm lift and discomfort > 30°, Pearson´s product-moment correlation coefficient = 0.46	− 2
Manchester (1988)	Music students who consulted the author’s student health service due to playing-related musculoskeletal disorders of the upper limb, USA	132	–	Instrument playing (plus gender, instrument group)	“Upper extremity problems brought by playing an instrument”, especially hand disorders	“Incidence” of hand disorders 8.5 per 100 music students per year (m: 5.7, f: 11.5), keyboard instruments: 13.2, strings: 9.6, wind instruments: 3.9)	− 11
Manchester and Lustik (1989)	Music students who consulted the author’s student health service due to playing re-related musculoskeletal disorders of the upper limb, USA	49	–	Studying music	“Performance-related hand problems”	50% of follow-up patients were symptom-free, 34% had a complaint reduction, 16% had persistent complaints	− 8
Manchester and Flieder (1991)	Music students who consulted the author’s student health service due to playing re-related musculoskeletal disorders of the upper limb, USA	114	–	Studying music (plus gender, instrument group)	“Performance-related hand problems”	“Incidence” of hand problems 8.5 (range 8–9.5) per 100 music students per year (m: range 4.9–7.2, f: range 9.5–12.1, *p* < 0.04, for keyboard instruments and strings higher than for wind instruments, *p* < 0.01)	− 11
Nusseck et al. (2017)	Music students, 5 universities of music, Germany	288	–	Studying music, preventive activities and health-related courses at universities	“Playing-related health problems” (mixed somatic and psychological)	No differences between the universities, prevalence during “course of study”: 29% in 1st year, 42% in 2nd year, among students with complaints somatic complaints 75% 1st year, 64% 2nd year, 66% 3rd year, 65% 4th year; risk/preventive factors: 41% students without complaints and 73% of students with complaints attend preventive courses in 1st year (Chi^2^(285) = 23.615, *p* < 0.001), 84% students without complaints and 68% students with complaints attend preventive courses in 2nd year (Chi^2^(134) = 4.822, *p* = 0.028), no stat. sing. difference 3rd year 70% vs. 85%, or 4th year 77% vs. 77%, high drop out between years	10
Piatkowska et al. (2016)	Music students, Poland	45 (15 violin, 15 cello, 15 piano)	–	Instrument playing: 1. group violin, 2. group cello, 3. group piano	“Cervical pain” by VAS (0–10 cm), disability by NDI (inter alia)	Moderate degree of disability and pain in all the groups, more disability and pain in the cellists, slightly lower in the violinists and the lowest in the pianists, pain stat. sign. lower (*p* = 0.04) 12 weeks after baseline in pianists compared to cellists	1

*f* female, *m* male, *NDI* Neck Disability Index, *stat. sign.* statistically significant, *QA* quality assessment, *VAS* visual analog scale

#### Quality assessment of the cohort studies

The quality assessment scores of the six cohort studies were between − 11 and 10 out of 16 possible points, mainly due to significant methodological concerns in most of the studies. Only three of the studies (Fjellman-Wiklund and Sundelin 1998; Nusseck et al. 2017; Piatkowska et al. 2016) had clearly defined study populations. None of the studies reported sample size calculations, and only one study (Nusseck et al. 2017) prospectively measured risk factors. However, three studies applied validated outcome measurements (Nusseck et al. 2017; Piatkowska et al. 2016; Kuorinka et al. 1987; Fjellman-Wiklund and Sundelin 1998).

### Cross-sectional studies

We found 62 cross-sectional studies (see Table 3).

**Table 3:** Characteristics and results of the included studies, cross-sectional studies

Author (year)	Population	Cases (*n*)	Control (*n*)	Exposure	Outcomes	Results	QA (*x*/16 points)
Abréu-Ramos and Micheo (2007)	Orchestra musicians “Puerto Rico Symphony Orchestra”, Puerto Rico	75	–	Instrument playing (plus instrument group, gender, age)	“MSKPs” (musculoskeletal problems) that affect playing	Lifetime prevalence MSKPs 81% (female 87,5% vs. male 97.7%); most common back pain (75%); most common in lower strings (93%) and percussionists (100%), in younger (22–29 years, 83%) and older (50–61 years, 91%) populations; female sex, age, instrument played are risk factors	− 3
Ackermann and Adams (2003)	Violinists and violists (students and orchestra musicians), Australia	32	–	Instrument playing (plus anthropometric measurements [length and ROM])	“Performance-related pain”: pain in different body sites related to playing	Lifetime prevalence “Performance-related pain” 88%, most common left upper extremity (69%), thoracic spine (63%), right upper extremity (53%) and cervical spine (44%); 4 greater ROM of the left hand compared with the right; left hand anthropometrics are not related to pain but anthropometrics of the right arm seems to be a risk factor	− 5
Ackermann et al. (2011)	Collegiate flute players, “Sydney Conservatorium of Music”, Australia	20	–	Flute playing	“PRMDs” (performance-related musculoskeletal disorders): prevalence and characteristics	Lifetime prevalence of 43 different PRMDs 95%, (37% lasting < 3 months, 63%, lasting for > 3 months), most common location: upper extremity; most common symptom: pain	− 9
Amorim and Jorge (2016)	Violinists (professional and students in higher education training), Portugal	93	–	Violin playing	“TMD” (temporomandibular disorder) in relation to MPA level, instrument practice time, chinrest type, gender and age	Prevalence TMD 58% (*n* = 50), slightly higher in females (55% vs. 53%), younger players aged ≤ 30 yrs. (61% vs. 55%), musicians with < 20-year experience (55% vs. 52%), practice < 22 h/week (54% vs. 53%) over the tailpiece chinrest model (57% vs. 50% in Dresden chinrest and 44% other models); risk factor: association between the prevalence of TMD and high MPA levels (*p* < 0.001), the most anxious violinists 6 times (95% CI 2.51–15.33; *p* < 0.001) more likely to report TMD symptoms vs. least anxious violinists	3
Arnason et al. (2014)	Music students (classical vs. rhythmic music), Iceland	74	–	Instrument playing (plus classical vs. rhythmic music, instrument group, gender)	“PRMD” (playing-related musculoskeletal disorder): prevalence and severity of musculoskeletal disorders	Lifetime prevalence PRMD 62% (70% classical vs. 39% rhythmic music; 61% in female, 39% in male, n.s. differences between instrument groups); prevalence PRMD last 7 days 40% (n.s. differences classical vs. rhythmic music)	− 7
Barton et al. (2008)	Music students, USA	97	–	Instrument playing (plus gender, instrument group)	“Physical symptoms”, disabilities by DASH; pain in any location	Average DASH score per person 6.62/100 (SD 8.69; range 0.0–45.0) f 8.58, m 4.67); strings higher mean DASH score than brass (mean difference = 7.98, *p* < 0.01) and woodwinds (mean difference = 6.50, *p* < 0.01); 65% current pain, (stat. sign. more f, sign diff. between instrument groups)	− 5
Berque et al. (2016)	Permanently employed classical orchestra musicians from 3 orchestras, Scotland	101	–	Instrument playing (plus gender, age, instrument group, playing professionally in an orchestra)	“PRMPs” (playing-related musculoskeletal problems), pain intensity and pain interference on function and psychosocial variables by Musculoskeletal Pain Intensity and Interference Questionnaire for Musicians (MPIIQM)	Prevalence PRMPs: lifetime 77.2%, 12 months 45.5%, point prevalence 36.6%, among PRMP 43% pain in ≥ 3 locations, most commonly the right upper limb, neck, left forearm and elbow, variations between instrument group; mean pain intensity in musicians with PRMP 12.4 ± 7.63 (out of 40), mean pain interference score 15.2 ± 12.39 (out of 50), increasing with the number of reported pain locations (*p* = 0.044); mean age in musicians with PRMP almost 5 yrs. older vs. non-PRMP (*p* = 0.029), average number of years of playing professionally in an orchestra with musicians with PRMP having almost 5 yrs. more vs. non-PRMP (*p* = 0.046)	3
Blackie et al. (1999)	Piano students, USA	16	–	Piano playing	Playing-related “injuries”/overuse: incidence, i.a.	Prevalence 93%, 27 playing-related injuries reported (66% in hand and wrist, among them 21% pain or discomfort impairs activities other than piano)	− 5
Chan et al. (2013)	Orchestra musicians (symphony orchestra), Australia	83	–	Instrument playing	Musculoskeletal “injuries” incl. “PRMDs” (performance-related musculoskeletal disorders), i.a.	99 consultations (83 individuals) among them 66% of injuries classified PRMDs (26% acute, 46% chronic recurring, 28% chronic; 93% currently affect playing, 94% preventable); most common locations shoulder (22%), neck (18%), upper back (18%), hand (8%)	− 11
Crnivec (2004)	Orchestra musicians “Slovene Philharmonic Orchestra” vs. marketing-workers “Philip Morris Enterprise”, Slovenia	70	28	Instrument playing	“Performance-related musculoskeletal disorders”, i.a.	Musculoskeletal disorders most common health impairment of musicians, almost 6 times higher than control group (147 vs. 25), most high in double bass and cello players	− 9
Cruder et al. (2017)	Music students at conservatories, Switzerland and UK	158	–	Instrument playing, instrument playing in symmetric playing position (SPP), asymmetric playing position (APP) and singing/voice	“Pain location and pain extent”, prevalence of pain according to SPP vs. APP vs. voice; pain intensity in any location (digital pain drawings); disabilities of the arm, shoulder, hand	Prevalence of pain 79.7% (*n* = 126) musicians, similar prevalence in musicians with SPP (75%, *n* = 56) and APP (78,2%, *n* = 78), highest prevalence in singers (95,8%, *n* = 24), higher prevalence in the neck and shoulders, lower back and the right arm; mean percentage of pain extent 3.1% ± 6.5%; mean QD and optional QD performing arts module score higher for musicians with pain vs. no pain (*p* < 0.001); positive correlation between the QD score and pain extent ((*p* ≤ 0.001), mean number of practice hours lower for people with pain (*p* = 0.002)	1
Davies and Mangion (2002)	Musicians (classical and non-classical), Australia	240	–	Making music (plus gender, years playing, instrument group, ergonomic problems, warm-up, rest-break provision, playing load, noise disturbance, playing-related stressors, health status, exercise behavior, playing-related muscle tension, preventive behaviors, training in prevention)	“Playing-related musculoskeletal pain and symptoms”: prevalence and severity	Lifetime prevalence: 51% with occasional recurrences; 22% with regular recurrences, 8% with permanent complaints, 7% without recurrence; 7% never had playing-related musculoskeletal pain and symptoms; positive risk factor string instrument, fewer years of playing, high muscle tension, high stress, association with frequent preventive behaviors	− 7
De Smet et al. (1998)	Pianists vs. volunteers, Belgium	66	66	Piano playing (plus hypermobility, hand size, playing habits and sports activity)	“Overuse syndrome” (musculoskeletal disorders of upper extremity)	Prevalence 45% in pianists vs. 8–12% in controls; wrist most common localization in pianists; risk factors: n.s. difference between pianists with or without overuse syndrome regarding playing habits, sports activity, hypermobility; hand size sign. height in male pianists without overuse syndrome	− 11
Eller et al. (1992)	Instrumentalists and opera singers “Royal Theatre”, Denmark	91/51	–	Making music (plus instrument playing vs. singing)	Symptoms from the musculoskeletal system, i.a.	Prevalence of symptoms equal in both groups, instrumentalists stat. sign. more symptoms upper extremity (OR 3.1, 95% CI 1.0–9.5, *p* = 0.047), but less in articulations of lower extremity (OR 0.2, 95% CI 0.07–0.61, *p* < 0.005); n.s. difference in low back pain	1
Engquist et al. (2004)	Orchestra musicians vs. actors, Sweden	103	106	Instrument playing (plus instrument group, gender, age)	“Musculoskeletal pain”: prevalence and intensity, VAS (0–20)	Point prevalence pain 61% vs.71% (OR 0.6); 12-month prevalence chronic pain 47% vs. 51% (OR 1); neck and shoulder most common, strings most often; no group difference regarding intensity of pain or risk factor gender	− 5
Gasenzer et al. (2017)	Orchestra musicians, 132 German cultural orchestras, Germany	740	–	Making music	“Chronic pain”: prevalence; degree of impairment, pain locations per instrument group, i.a.	Prevalence 66% (*n* = 490) current or recurring pain, 64% (*n* = 470) continuous pain or pain > 3 months, most frequent in back (70%), shoulders (68%), neck (64%), hands and wrists (40%); 27% pain with high degree of impairment (Korff scale); risk factor high strings highest rate in chronic shoulder pain i.a.; mean pain maximal intensity 6.0 (SD ± 2.4)	− 5
Fjellmann-Wiklund et al. (2003)	Music teachers, Sweden	208	–	Employed music teachers (plus Physical activity during leisure time, perceived health, physical work environment, psychosocial work environment)	“Musculoskeletal discomfort in the neck–shoulder region”: prevalence, SNQ	12-month prevalence 82%, most common neck (59%), shoulder (55%), lower back (45%); risk factor female stat. sign. more symptoms in neck, shoulder, upper back; in female: strongest risk factors associated with neck–shoulder discomfort: high psychological demands (OR 6.0, CI 1.1–32.4), teaching many schools (OR 4.8, CI 1.0–24.4); in male: lifting (OR 8.7, CI 2.1–34.8), playing the guitar (OR 6.0, CI 1.5–23.6), low social support (OR 3.1, CI 1.0–9.7)	5
Fotiadis et al. (2013)	Orchestra musicians (symphonic orchestra) “Athens and Thessaloniki State Symphony Orchestra”, Greece	147	–	Instrument playing (plus gender, age, instrument group, daily instrument practice)	“Musculoskeletal disorders”: prevalence	Lifetime prevalence 82%, 66% with considerable influence on performing ability; risk factors: neck/shoulder in female stat. sign. more frequent; shoulder in string instrumentalists stat. sign. More frequent than in brass/woodwind instrumentalists; wrist/hand in musicians > 60 years. stat. sign. more frequent, hours of practice per day is a criterion for occurrence of musculoskeletal disorders	1
Fry (1986)	Orchestra musicians, Australia, USA, England	485	–	Instrument playing	“Overuse syndrome”: pain and prevalence with severity	Prevalence 64%, most common localization hand/wrist (41%), neck (38%), shoulder (35%) and LWS (26%)	− 9
Gohl et al. (2006)	University pianists “Belmont University School of Music” and “Vanderbilt University Blair School of Music”, USA	19	–	Piano studies	“Median and ulnar neuropathies” in either upper extremity	Prevalence 16% electrodiagnostic evidence of early median neuropathy at or distal to the wrist, no further signs of neuropathy	− 1
Heikkilä et al. (2012)	Orchestra musicians “Sinfonia Lahti” and “The Finnish Radio Symphony Orchestra”, Finland	73	–	Instrument playing (plus instrument group, age, gender, stress, night bruxism, day bruxism, sleep disturbances, physical exercise)	“Symptoms of TMDs and facial pain”	1-month prevalence 56%; no difference in instrument groups; risk factors: sleep disturbances and night bruxism seem to increase TMD’s, TMDs diminish with age	3
Heredia et al. (2014)	Pop-musicians “Orquesta Buena Vista Social Club and Supporting Bands”, Kuba	36	–	Making music	“Musculoskeletal conditions”	12-month prevalence 37%	− 9
Hodapp et al. (2009)	Orchestra musicians (opera and symphonic orchestra vs. amateur orchestra), Germany	122	28	Instrument playing (plus professional vs. amateur musicians, work complexity, working conditions, scope of action, social stressors, number of performances and rehearsals, i.a.)	“Bodily complaints” including musculoskeletal complaints, i.a.	Prevalence musculoskeletal complaints more in professional musicians than in amateurs (*M* 5.24, SD 4.76 vs. *M* 2.71, SD = 2.21; *p* < 0.001); risk factors work stressors and number of performances correlate pos. with musculoskeletal complaints	− 5
Kaufman-Cohen and Ratzon (2011)	Orchestra musicians (string and wind players), Israel	59	–	Instrument playing (plus biomechanical, environmental, psychosocial and personal risk factors, individual playing characteristics)	“PRMD” (playing-related musculoskeletal disorders): musculoskeletal pain and functional impairment during last years, severity, SNQ, DASH, RULA	12-month prevalence PRMPD min. 1 body region 83%, > 1 body region 73%; biomechanical and postural loading, instrument weight, risk factors perceived physical environment, average playing hours per week, gender and warm-up’s correlate with PRMDs	5
Kim et al. (2012)	Traditional Korean string instrument players, Korea	86	–	Instrument playing (plus type of instrument, age, height, weight, BMI, years of career, gender, practice habits, exercise intensity, hobby styles, drinking and smoking habits, stretching)	“PRMDs” (playing-related musculoskeletal disorders): musculoskeletal disorders	Prevalence each > 50% in forearm, neck, back, shoulder, upper arm, wrist and knee; risk factors: various stat. sign. correlations between demographic variables and musculoskeletal disorders depending on type of instrument	− 7
Ackermann et al. (2012)	Orchestra musicians, Australia	377	–	Instrument playing (plus instrument group)	“PRMDs” (performance-related musculoskeletal disorders, performance-related musculoskeletal pain disorders), musculoskeletal disorders	Lifetime prevalence 84%, point prevalence 50%, most common localization: lower back (14.1%), upper back (11.7%) and shoulder/upper arm (11.1%); localization varies according to instrument group	1
Kenny and Ackermann (2015)				Instrument playing (plus gender, age, depression, performance anxiety, social phobia)		Lifetime prevalence 84%, point prevalence 50%, risk factor: female, positive correlation between performance anxiety and intensity of PRMD and between depression and intensity PRMD	
Kenny et al. (2016)	Orchestra musicians, Australia	378	–	Instrument playing in different orchestra types: pit, stage and combined stage and pit	“PRMDs” (performance-related musculoskeletal disorders), “physical and mental health indicators”, VAS pain, i.a.	Lifetime prevalence pain or injuries caused by playing 81–95%, lifetime prevalence pain or injuries interfering with playing 85–90%, point prevalence pain or injury 46–56%, n.s. difference between 3 types of orchestras regarding lifetime or point prevalence of PRMD; 42% pit, 23% stage and 28% stage/pit musicians had time off work due to physical pain or injury last 18 months, n.s. difference in number of days taken	− 1
Kochem and Silva (2017)	Violinists, Brazil	106	–	Instrument playing	“PRMDs” (playing-related musculoskeletal disorders): prevalence by SNQ last 12 months and last 7 days, associated factors, disabilities by DASH	12-month prevalence: 87%, 1-week prevalence 77%, prevalence of PRMDs responsible for the temporary interruption of musical activity: 8%; most frequently affected in both time periods neck, thoracic area, right and left shoulders, left wrist/hand Prevalence > 50% for dysfunctional upper limbs according to the DASH optional module; mean DASH score 10.6 points (SD 8.6), optional DASH music module mean score of 17.6 points (19.8) Risk factors: women more likely PRMDs (OR 4.4, CI 1.9–10.0, *p* < 0.001); older musicians more likely pain in last 7 days (OR 3.3, CI 5.1–10.97; *p* = 0.04) and higher scores on DASH (OR 1.8, CI 1.1–3.1; *p* = 0.01); also associated with PRMD: body mass index, practice hours per week, final DASH score, i.a.	3
Kok et al. (2015)	Music students vs. medical students, Netherlands	83	494	Studying music (plus instrument group)	“CANS” (complaints of arm, neck, and/or shoulder not caused by a systemic disease or acute trauma) and gender	Point prevalence in music students vs. medical students (47% vs. 18%, *p* < 0.001), 12-month prevalence in music students vs. medical students (81% vs 42%, *p* < 0.001), chronic CANS in music students vs. medical students (36% vs. 10%, *p* < 0.001); music students: more complaints per anatomic localization and more localizations, music students more severe influence of CANS on daily functioning (5.0 vs. 3.1, *p* < 0.001); most common neck in 46% music students and 27% medical students (*p* = 0.001); risk factor: higher prevalence of CANS in female music students vs. male music students (84% vs. 71%, *p* = 0.212), higher prevalence of CANS in bachelor’s students vs. master’s students (85% vs. 55%, *p* = 0.018)	− 1
Kok et al. (2013)					“Musculoskeletal complaints”	Point prevalence in music students vs. medical students (63% vs. 43%, *p* = 0.001, OR 2.25); 12-month prevalence in music students vs. medical students (89% vs. 78%, *p* = 0.019, OR 2.33); in total more complaints in upper body half	− 1
Kovero and Könönen (1995)	Violinists and violists “Helsinki Philharmonic Orchestra” vs. patients of Institute of Dentistry, University of Helsiki, Finland	26	26	Professional playing violine or viola, weekly playing time	“Signs and symptoms of TMD” or radiological abnormalities in the temporomandibular condyles	Frequency of temporomandibular pain 27% (musicians); musicians have more signs (but not symptoms or radiological abnormalities) of TMD than controls; pos. correlation between weekly playing time and symptoms of TMD, i.a.	− 7
Leaver et al. (2011)	Orchestra musicians (symphonic orchestra), England	243	–	Instrument playing (plus physical activities at work, psychosocial factors of working environment, performance anxiety, instrument group, mental health, smoking, age, gender)	“Musculoskeletal pain”: prevalence and impact, relation to playing conditions, including instrument category, i.a.	12-month prevalence of regional pain 86%, impairing pain 41%, 1-month prevalence of regional pain 71%; risk factors: positive correlation with high somatizing scores (OR 2.5) and female gender; no correlation with performance anxiety; variation of disorders according to instrument group	5
Lima et al. (2015)	Orchestra violinists, Belo Horizonte, Brazil	18/33	–	Instrument playing	“Functional disorders of the musculoskeletal systems”; VAS pain, BPSF	Prevalence of muscular pain 78%(*n* = 14) of muscular fatigue 33% (*n* = 6), prevalence of reported diagnoses by participants (*n* = 15 respondents): tendinitis in the upper extremity 28% (*n* = 5), pain cervical spine 17% (*n* = 3), back pain 17% (*n* = 3) i.a., VAS pain (0–10): average 5.6; Prevalence according to Wisconsin’s Pain Inventory (short form) pain in: lumbar spine 24% (*n* = 8); shoulders 21% (*n* = 7), forearms and hands 18% (*n* = 6); cervical spine 12% (*n* = 4); legs and feet 6% (*n* = 2); and headaches 6% (*n* = 2); prevalence of pain interfering with work (scale of 0–10): average rating of 7.1 (median 8.0)	− 9
Logue et al. (2005)	Cello students “Belmont University School of Music” and “Vanderbilt University Blair School of Music”, USA	14	–	Cello studies	“Median and ulnar neuropathies”: prevalence, either upper extremity, physical examination, EMG	No evidence of median or ulnar neuropathy	1
Marques et al. (2003)	Guitarists (participants of classes about prevention of overuse), Spain	64	_	Guitar playing, classical guitar vs. flamenco guitar	“Overuse syndrome”: prevalence	Point prevalence 75%, classic guitarists vs. flamenco guitarists: 66% vs. 88%	− 5
Fishbein et al. (1988)	Orchestra musicians, USA	2212	–	Instrument playing	“Musculoskeletal problem”, i.a.	Prevalence (all localizations) 82%, most common localizations: shoulder (20%), neck (22%) and lower back (22%)	− 7
Middlestadt and Fishbein (1988)				Instrument playing (plus perceived occupational stress)		Risk factor: significant relationship between number of musculoskeletal problems and perceived occupational stress	
Middlestadt (1990)	Orchestra musicians, USA	2212	–	Instrument playing (plus gender)	“Musculoskeletal problem”, i.a.	Prevalence (all localizations) 82%, prevalence of severe reported musculoskeletal disorders: 59% (risk factor female vs. male 70% vs. 54%)	− 7
Middlestadt and Fishbein (1989)	Orchestra musicians: subgroup string players, USA	1378	–	Instrument playing (plus instrument group)		Prevalence of severe reported musculoskeletal problems: 66% in string players (compared to woodwind players with 48% and brass players with 32%), most common shoulder, neck and lower back; risk factor sting playing: prevalence stat. sign. higher in females vs. males	
Miller et al. (2002)	Music students “Royal Northern College of Music” (string players, keyboard players) vs. nonmusicians from various hospital departments, England	92	64	Instrument playing (plus previous injury, years at college, instrument group, years playing an instrument, duration of practice periods, age, gender, various anthropometric parameters, i.a.	“Upper limb pain and dysfunction”, anatomical abnormalities	Prevalence: music students five times more likely for upper limb pain; risk factor previous injuries, years at college, instrument group, years studying instrument and practice period stat. sign. correlate with upper limb pain; anatomical abnormalities (and variations) 72% vs. 59% (n.s.)	− 5
Mishra et al. (2013)	Indian tabla players, India	85	–	Tabla playing (folded-knee sitting posture)	“PRMDs” (playing-related musculoskeletal disorders): prevalence by NMQ	Prevalence in low back 73%, right shoulder 60%, neck 54%, left shoulder 51%, upper back 45%, right knee 45%, left knee 46%, VAS scores (no scale reported) for intensity of discomfort in different body parts 3.42 (low back) to 1.63 (right knee)	− 7
Molsberger (1991)	Orchestra musicians “Deutsche Oper Berlin” and “Düsseldorfer Symphoniker”, Germany	100	–	Instrument playing	“Disorders of the locomotor apparatus”, i.a.	Prevalence 75%, most common: neck 35%, other parts of spinal column 16%; average disease duration 61.3 months, prevalence of impairing disorders: 45%.	− 7
Monaco et al. (2012)	Orchestra musicians “Teatro dell’ Opera”, Italia	65	–	Instrument playing	“PRMDs” (playing-related musculoskeletal disorders), DASH	Prevalence musculoskeletal disorders in daily life (defined as ≥ 15 points in DASHi) 28%, prevalence complaints during playing 51% (DASH additional module)	− 9
Moore et al. (2008)	Music students (upper string players) vs. control group, USA	10	18	Instrument playing (plus years instrument played, weekly playing time, rest time, shoulder assessment data)	“Predisposing factors for shoulder impingement syndrome” (Signs and symptoms of shoulder impingement)	Prevalence of pain while playing 70%, most common shoulders (left 50%, right 30%) and neck (left 40%, right 20%). 30% shoulder impingement vs. 0% in controls (stat. sign. correlation); risk factors: n.s. correlation with years instrument played, weekly playing time, rest time, shoulder assessment data)	− 1
Navia Alvarez et al. (2007)	Orchestra musicians, Spain	48	–	Instrument playing	“Neck pain syndrome”: prevalence	Lifetime prevalence neck pain 69%, 12-month prevalence 63%; prevalence last 7 days 27%; lifetime prevalence tingling in upper extremity 44%, lifetime prevalence loss of sensation or force 40%; most important risk factor: labor stress, no clear relation between instrument played and the years of professional activity, neither with the sex or age	− 9
Nyman et al. (2007)	Orchestra musicians, Sweden	235	–	Instrument playing with elevated arm position and daily playing time	“Neck–shoulder pain”	Point prevalence 26% in whole population; risk factors: neutral arm position, < 2 h per workday’: 9% vs. ‘neutral arm position, > 3 h per workday’: 19%; ‘elevated arm position, < 2 h per workday”: 30% vs. “elevated arm position, > 3 h per workday”: 35%; higher odds for subjects with neck/shoulder pain in the groups “elevated arm position, < 2 h per workday” [OR 4.15 (1.30–13.22)], and “elevated arm position, > 3 h per workday” [OR 5.35 (1.96–14.62)] compared to the group “neutral arm position, < 2 h per workday”	5
Paarup et al. (2011)	Orchestra musicians (symphonic orchestra) vs. representative sample from the Danish workforce from The Danish Working Environ- ment Cohort, Denmark	342	5436	Instrument playing (plus instrument group, gender)	“Perceived musculoskeletal symptoms”: prevalence	12-month prevalence for disorders in min. 1 region: female 97% and male: 83% (OR 6.5), risk factor: woodwind players stat. sign. lower risk than upper string players; symptoms more frequent and lasted longer in musicians than in general workforce	3
Papandreou and Vervainioti (2010)	Percussionists (in active musical activity and students), Greece	30	–	Instrument playing (plus age, main musical activity [student, orchestra musicians, music teacher, solist], practice hours)	“Musculoskeletal disorders”	Prevalence 32% in upper extremities, 20% in vertebral column	− 3
Raymond et al. (2012)	Classical orchestra musicians, USA	32	–	Instrument playing	“Occupational injury and illness”, i.a. musculoskeletal disorders	Lifetime prevalence: pain or stiffness in shoulders 94%, pain or stiffness in neck 91%, numbness and tingling in hand or arms 81%, low back pain 63%; Lifetime prevalence of diagnosed most common disorders: tendinitis 47%, musculoskeletal disorder 22%, carpal tunnel syndrome 16%	− 7
Rein et al. (2010)	Organists and pianists vs. controls with no work-related increased use of their feet, Germany	30/30	30	Instrument playing	“Work-related influences on functional ankle stability”	Organists have neither increased functional ankle stability nor increased ROM of their ankle joints in comparison to controls; Pianists have increased flexion of the ankle joint on both sides in comparison to organists and on the right side in comparison to controls	− 3
Rickert et al. (2012)	Cellists (orchestra musicians vs. students), Australia	47/25		Cello playing (plus playing habits, lifestyle factors and gender)	Right “shoulder injury levels and causes”: frequency and severity of reported “PRMDs”; physical data on shoulder strength, ROM and signs of injury	Mean age students 19 years (17–26), orchestras 42 years (24–63); 18-month prevalence of reported disorders 89% (orchestra) vs. 56% (students); disorders right shoulder 42% (orchestra) vs. 20% (students); risk factor: prevalence PRMDs stat. sign. higher in female	1
Sakai (2002)	Pianists and piano students, seeking orthopedic consultation with the author, Japan	200	–	Piano playing	Differential diagnoses of “hand pain” due to “overuse”, cause of overuse (keyboard technique at the time of the onset, practice time)	Tenosynovitis and tendinitis (*n* = 56), enthesopathy (*n* = 49), muscle pain (*n* = 38), finger joint pain (*n* = 22), cubital tunnel syndrome (*n* = 8), carpal tunnel syndrome (*n* = 2), neck and scapular pain (*n* = 7), focal dystonia (*n* = 18); octaves and chords 74% of techniques practiced at onset; time of piano playing day before pain 3.7 (range 2–13) hours	− 13
Schäcke et al. (1986)	Orchestra musicians (opera orchestra), Germany	109	–	Instrument playing (plus age and years playing)	“Musculoskeletal complaints”	Prevalence neck pain 65% (among them 40% muscle tension), low back pain 44% (among them 24% muscle tension), thoracic spine pain 22%, complaints in shoulder and arm 18%; risk factors: musicians with musculoskeletal complaints are older (46 ± 10 years vs. 37 ± 8 years) and have more years playing (23 ± 10 vs. 14 ± 8 years) than musicians without musculoskeletal complaints	− 9
Shields and Dockrell (2000)	Piano students, Ireland	159	–	Piano playing (plus practice time per day and week, gender)	“Playing-related injuries”, interrupting playing > 48 h	Lifetime prevalence 26% (among them 37% at the wrist and 15% at the fingers; most common symptom was pain with 98%), point prevalence 7%, risk factors: n.s. difference in playing time or gender	− 1
Sousa et al. (2016, 2017)	Professional orchestra musicians, 3 orchestras, Portugal	112	–	Instrument playing, instrument group: wind instrument vs. string instrument	“PRMDs” (playing-related musculoskeletal disorders)	Point prevalence 62.5% (among them: shoulder 27%, cervical 27% and lumbar region 24%; risk factors: string players more frequently affected by PRMDs (67.6% vs. 54.1%), pain intensity n.s. higher in wind players than in string players, pain in instrument groups listed	− 3
Stanhope et al. (2014)	Woodwind students, Australia	14	–	Woodwind playing	“PRI” (playing-related injuries): musculoskeletal symptoms, that prevent musicians from playing at their normal level	Lifetime prevalence 62% (*n* = 8) (back and upper extremity most common), point prevalence 38% (neck/shoulder and upper extremity most common)	− 5
Steinmetz et al. (2007, 2012)	Music students vs. pedagogy students, Germany	36	19	Music studying	“PRMD” (playing-related musculoskeletal disorders): pain and discomfort playing the instrument and musculoskeletal dysfunctions	Lifetime prevalence PRMD 81% (music students), point prevalence pain (pain during examination) 0%, in physical examination more musculoskeletal dysfunctions in music students (8.39/person vs. 4.37/person)	− 3
Steinmetz et al. (2003, 2006)	Violinists, Germany	31	–	Signs and symptoms of craniomandibular dysfunction (CMD), muscular load masticatory and neck muscles with and without occlusal splints	Muscular load masticatory and neck muscles during violin playing (supposed to be a cause/predictor of overuse syndrome) via surface EMG with and without occlusal splints, frequency of CMD, overuse syndrome measured via questionnaire	Lifetime prevalence pain during violin playing 81%, overuse syndrome 74%, prevalence pain (in period around examination) 39%, point prevalence pain (pain during examination) 0%; oral splints decrease stat. sign. the muscular load (*p* ≤ 0.001 for masseter; *p* ≤ 0.01 for temporalis and sternocleidomastoid muscles; and *p* ≤ 0.1 for trapezius muscle, n.s. decrease in extensor muscles)	− 9
Steinmetz et al. (2014)	Classic orchestra musicians, Germany	408	–	Instrument playing according to instrument group (plus symptoms of CMD)	“CMD”: frequency and its association with musculoskeletal pain	3-month prevalence playing-related pain in teeth or jaw 19–47% and in TMG pain in 15–34%, point prevalence pain in the face indicating a painful CMD 6–10%, violin players highest prevalence of all CMD symptoms; musicians reporting with orofacial pain are 4.8 times more likely to report musculoskeletal pain in other localizations	− 1
Steinmetz et al. (2015)	Classic orchestra musicians, Germany	408	–	Instrument playing according to instrument group	“Playing-related musculoskeletal pain”: Frequency and intensity (NRS), with regard to instrument	Lifetime prevalence 90% (pain in neck/cervical spine 73%, left shoulder 55%, left wrist 55%, right shoulder 52% and lumbar spine 51%), mean pain intensity 3.7 (SD 1.95), 3-month prevalence 63%, point prevalence 9%; risk factor female gender and stage fright were proven to be predictors for musculoskeletal pain	− 1
Steinmetz et al. (2016)	1. Music students and professional violin and viola players violin/viola players with neck pain vs. 2. music students and professional violin/viola players without neck pain vs. 3. pain-free non-musicians, Germany	12 vs. 21	21	Instrument playing	“Flexor muscle behavior in violin/viola players with and without neck pain”, pain VAS (0–10 cm), disability NDI, PSFS, EMG i.a.	Pain intensity in violinists with pain 5.1 (SD 2.5), disability (NDI) group 1: 24.7 (SD 4.7), group 2: 5.3 (SD 5.4), group 3 1.9 (SD 3.4), risk factor violin/viola players with neck pain had greater normalized SCM EMG amplitudes during craniocervical flexion test than the pain-free musicians and non-musicians (*p* < 0.05)	1
Wahlström Edling and Fjellman-Wiklund (2009)	Music teacher (Instrumental teacher), Sweden	47	–	Physical workload by instrument playing, (plus posture and playing time per week)	“Musculoskeletal disorders”, SNQ	12-month prevalence 77% (lower back 49%, neck 47%, upper back 32%, shoulder 28%); risk factors: more in females, asymmetric playing posture correlates stat. sign. with number of more musculoskeletal disorders, n.s. correlation to playing time	− 1
Woldendorp et al. (2016)	Professional and professional student double bassists and bass guitarists, Netherlands	141	–	Instrument playing (bass guitarists vs. double bassists) with postural stress, bowing style in double bassists	“Musculoskeletal complaints”, intensity of pain (NRS, 0–10), self-reported functioning	3-month prevalence 74% (most frequently in back and neck, up to 55% in bassists playing both instruments), 3-month prevalence of playing impairing complaints 43%, pain intensity mostly mild (NRS ≤ 3) last week, risk factors: no association between complaints and the playing position of the left shoulder area in double bassists (*p* = 0.30), the right wrist area in the bass guitarists (*p* = 0.70), the right wrist area for the German vs. French bowing style (*p* = 0.59), long-lasting exposures to postural stress were not associated with musculoskeletal complaints	3
Woldendorp et al. (2017)				Instrument playing (work load bass players: multi-vs. mono-instrumentalism)	“Musculoskeletal complaints”	Risk factors: 3-month prevalence of musculoskeletal complaints in the neck, back, right shoulder area and both wrist areas did n.s. differ between bass guitarists vs. double bassists, likelihood of musculoskeletal complaints in the left shoulder area higher in multi-instrumentalists vs. mono- instrumentalists (OR 0.30, 95% CI 0.119–0.753, *p* = 0.010), no protective effect of multi-instrumentalism against musculoskeletal complaints	
Yeung et al. (1999)	Orchestra musicians, Hong-Kong	39	–	Instrument playing (plus gender, professional life years, starting age, hours practice per week, breaks during practice sessions, warm-up’s, regular exercises, trauma unrelated to music playing)	“PRMC’s” (playing-related musculoskeletal complaints)	12-month prevalence 64%; risk factors: less professional life years and lack of regular exercises correlate to PRMC’s	− 1
Yoshimura et al. (2006)	Piano students “University of North Texas”, USA	35	–	Instrument playing (plus age, age started piano playing, years of private lessons, height, weight, BMI, various anthropometric parameters and elements of performance)	“Piano-related pain”: musculoskeletal pain, i.a.	Finger joint mobility, particularly right 3–4 span, is a risk factor for piano-related pain	− 3

*APP* asymmetric playing position, *BMI* body mass index, *BPSF* Brief Pain Short Form, *CI* confidence interval, *CMD* craniomandibular dysfunction, *DASH* Disabilities of the Arm, Shoulder and Hand, *EMG* electromyography, *f* female, *m* male, *M* mean, *MPA* music performance anxiety, *NDI* Neck Disability Index, *NMQ* Nordic Musculoskeletal Questionnaire, *NRS* Numeric Rating Scale, *n.s.* not statistically significant, *OR* odds ratio, *PSFS* patient-specific functional scale, *QA* quality assessment, *QD* QuickDASH, *stat. sign.* statistically significant, *ROM* range of motion, *RULA* Rapid Upper Limb Assessment, *SCM* sternocleidomastoid, *SD* standard deviation, *SNQ* Standardized Nordic Questionnaire, *SPP* symmetric playing position, *TMD* temporomandibular disorder, *VAS* Visual Analog Scale

#### Outcome measures used in cross-sectional studies

MCDs in the cross-sectional studies were measured with different instruments and used different definitions and questionnaires that to our knowledge, were often not validated (Abréu-Ramos and Micheo 2007; Ackermann et al. 2011; Arnason et al. 2014; Blackie et al. 1999; Chan et al. 2013; Crnivec 2004; Davies and Mangion 2002; De Smet et al. 1998; Fishbein et al. 1988; Fry 1986; Heredia et al. 2014; Hodapp et al. 2009; Kim et al. 2012; Kok et al. 2013; 2015; Kovero and Könönen 1995; Marques et al. 2003; Schäcke et al. 1986; Middlestadt and Fishbein 1989; Molsberger 1991; Papandreou and Vervainioti 2010; Raymond et al. 2012; Steinmetz and Möller 2007; Shields and Dockrell 2000; Sakai 2002).

#### Period of time investigated in cross-sectional studies

The time period of the measured MCD varied substantially between studies, ranging from whole lifetimes, years playing an instrument, the previous 1, 3, 12 or 18 months, the last 7 days or current complaints. For some publications, we could not find a specification of the exact time frame used by the study, f.e. (Lima et al. 2015; Blackie et al. 1999; Crnivec 2004; De Smet et al. 1998; Eller et al. 1992; Fishbein et al. 1988; Fry 1986; Schäcke et al. 1986; Hodapp et al. 2009; Kim et al. 2012; Kovero and Könönen 1995; Middlestadt 1990; Middlestadt and Fishbein 1988, 1989; Molsberger 1991; Papandreou and Vervainioti 2010).

#### Prevalence reported in cross-sectional studies

Due to substantial heterogeneity among the measured complaints, a comparison of prevalence and correlations to the MCD was not feasible. In the included studies, the prevalence of MCDs ranged from point prevalence 0% in one small study (Logue et al. 2005) to 12-month prevalence 97% in a study of female orchestra musicians (Paarup et al. 2011). However, within the four studies that received a quality assessment of 5, the 12-month prevalence ranged from 82 to 86% (Leaver et al. 2011; Kaufman-Cohen and Ratzon 2011; Fjellmann-Wiklund et al. 2003), with 26% of musicians reporting current complaints (Nyman et al. 2007).

#### Risk factors reported in cross-sectional studies

The four studies with the best quality assessments reported playing with an elevated arm position as a risk factor for neck-shoulder pain (Nyman et al. 2007). Neck-shoulder discomfort in female music teachers was correlated with high psychological demands and teaching at multiple schools, whereas in male music teachers, it was associated with lifting, playing the guitar and low social support (Fjellmann-Wiklund et al. 2003). In symphony orchestras, MCD tended to be more frequent among women, in musicians experiencing low mood and in those with high somatizing scores. Only weak associations were observed with psychosocial work stressors and performance anxiety (Leaver et al. 2011). In musicians who play string instruments, the odds of wrist/hand pain were 2.9-fold higher than for those who play wind instruments (Leaver et al. 2011). In contrast, another study found that in classical musicians, string musicians showed higher PRMD scores than woodwind and brass players. Furthermore, the study found a correlation with biomechanical risk factors, perceived physical environment risk factors, instrument weight and average number of hours played per week (Kaufman-Cohen and Ratzon 2011). However, cross-sectional studies have low validity for verifying risk factors such as exposure time, and the occurrence of outcomes cannot thus be properly measured.

#### Quality assessment of cross-sectional studies

The quality scores of the 62 cross-sectional studies were between − 13 and 5 out of 16 possible points, with significant methodological concerns existing in the studies. Only one study (Nusseck et al. 2017) utilized a time frame that could reliably measure the association between the exposure and outcome, although several studies explicitly looked for risk or predicting factors. No studies reported the blinding of outcome measures. Sample size calculations were reported in only two of the studies (Kochem and Silva 2017; Kaufman-Cohen and Ratzon 2011). Frequent concerns included the objectivity, reliability and validity of the outcome and exposure measurement tools. Often, no confounders were assessed, and only a few studies assessed at least some important confounders (Eller et al. 1992; Fjellmann-Wiklund et al. 2003; Leaver et al. 2011; Nyman et al. 2007; Kok et al. 2015; Nusseck et al. 2017; Piatkowska et al. 2016; Woldendorp et al. 2016, 2017).

### Intervention studies

#### Study designs and methods of the interventional studies included

Three of the 12 intervention studies were performed using pre–post study designs without a control group (Table 4). One of these three studies compared two different interventions, (strength vs. endurance training) stratified by instrument played, but further information on randomization was not provided (Ackermann et al. 2002b). The other nine interventions used controlled designs (Table 5). Seven of the 12 studies were randomized controlled trials. In one partially blind study, playing under the intervention or control condition was randomized (Ackermann et al. 2002a). In one study with a three-armed design, participants in the two intervention groups were randomized, but the control group was recruited separately (Khalsa et al. 2009). In one non-randomized study, allocation of the nine eligible orchestras followed geographical criteria; the six orchestras that were geographically closest to each other were selected as interventional orchestras, while the remaining three served as control orchestras (Brandfonbrener 1997). The second non-randomized study was a pilot study for a partially randomized trial that was conducted later. The pilot recruited its control group separately due to a low number of participants (Khalsa and Cope 2006; Khalsa et al. 2009). Two publications about Tuina treatment appeared to be drawn from the same study population, reporting immediate effects and effects occurring after 3 weeks (Sousa et al. 2015a, b).

**Table 4:** Characteristics and results of the included studies, pre–post studies without control groups

Author (year)	Population	Case (*n*)	Control (*n*)	Intervention	Outcome	Result	QA (*x*/15 points)
Ackermann et al. (2002a, b)	Music students “Canberra School of Music”, Australia	10/9	–	Group 1: 6-week strength training Group 2: 6-week endurance training	“PRMD’s” (performance-related musculoskeletal disorders): frequency and intensity, strength and endurance tests, i.a.	Changes in PRMD’s n.s.; stat. sign. strength gains in both exercise groups	4
Chan et al. (2014)	Orchestral musicians (symphonic orchestra), Australia	50	–	12-week exercise program by DVD, min 40 min of exercise per week	“PRMDs” (performance-related musculoskeletal disorders): frequency and intensity, i.a.	Reduction in the mean prevalence of PRMD from 3.3 (SD 2.9) to 2.1 (SD 2.1), in VAS (0–10) pain (95% CI − 2 to − 0.3 *p* < 0.01) and the mean intensity of PRMD from 2.9 (SD 2.4) to 1.9 (SD 1.9) in VAS (0–10) (95% CI − 1.8 to − 0.3, *p* < 0.01)	− 3
Steinmetz et al. (2009)	Musicians with craniomandibular dysfunctions (CMD), treated in outpatient practice of the authors, Germany	30	–	Time duration of treatment with oral splints (at least at night and during instrument playing) individually	“CMD”: symptoms, pain in multiple body regions	80% of participants reported a stat. sign. reduction in dominant symptoms, 20% of participants reported a decrease in the days unable to play, 40% of participants reported an increase in pain when not wearing the splint; mean pain in the upper extremity decreased from 3.0 to 0.9 (of max. 5); neck pain decreased from 3.0 to 2.4; pain in teeth/TMJ decreased from 1.7 to 1.0	− 4

*CI* confidence interval, *CMD* craniomandibular dysfunction, *n.s*. not statistically significant, *QA* quality assessment, *SD* standard deviation, *stat. sign.* statistically significant, *TMJ* temporomandibular joint, *VAS* visual analog scale

**Table 5:** Characteristics and results of the included studies, controlled clinical trials

Author (year)	Population	Cases (*n*)	Control (*n*)	Intervention	Randomization	Outcome	Results	QA (*x*/18 points)
Ackermann et al. (2002a, b)	Orchestra musicians: violinists, Australia	8	8 (same as cases)	Conditions for intervention: violin playing of 3 excerpts of pieces with taped scapula Conditions for control: violin playing of 3 excerpts of pieces without taped scapula	RT	“Pain” during playing, i.a.	N.s. change of pain during scapula taping	0
Brandfonbrener (1997)	Orchestra musicians, USA	177	138	1. Group: intervention with music didactic lectures and instructions for home exercises in strengthening and flexibility 15 min daily during 1 year 2. Group: control group without intervention	CT	“Musculoskeletal symptoms”	Musculoskeletal symptoms from test 1 (pre intervention), to test 2, to test 3 (end intervention) in intervention group: from 67 to 64% to 63%; in control group: from 54 to 42 to 48%, stat. sign. improvement from test 2 to 3; n.s. differences between groups	− 10
Damian and Zalpour (2011)	Musicians with non-specific shoulder–neck problems (students and professional musicians), Germany	13	13	1. Group: once a week trigger point treatment with radial shockwave therapy and physical therapy during 5 weeks (intervention) 2. Group: once a week physical therapy during 5 weeks (control group)	RT	“Shoulder and neck complaints” (pain and disability), VAS, SPADI, NPIDQ	Intervention group: pain measured by VAS declines during treatment (*p* = 0.000); shoulder pain and disability measured by SPADI improves from 16.2 (SD 8.6) to 8.4 (SD 6.35) (*p* = 0.014); neck pain and disability measured by NPIDQ: improves from 20.7 (SD 11.5) to 9.75 (SD 9.3) *p* = 0.016); in control group n.s. changes	0
De Greef et al. (2003)	Orchestra musicians (symphony orchestra) with PRMD’s (playing-related musculoskeletal disorders), Netherlands	25	28	1. Group: 45 min of exercise intervention following the GETSOM program (warm up, general exercising, specialized exercising in regard to instrument playing, cooling down and counseling) before each orchestra rehearsal during 15 weeks 2. Group: control group without intervention	RT	“PRMDs” (playing-related musculoskeletal disorders), WHQM	PRMD’s measured by WHQM improve from baseline to after intervention to 3-month follow-up in intervention group: from 98.5 to 97.5 to 96.8; stat. sign. (*p* < 0.05) and clinically relevant (*d* > 0.20) improvement in comparison to control group	− 2
Khalsa and Cope (2006)	Participants summer program “Tanglewood Music Center” (Boston Symphony Orchestra’s academy), USA	10	10	1. Group: yoga lifestyle intervention (during 8 weeks) 2. Group: no-practice-control group	CT	“PRMD’s” (performance-related musculoskeletal disorders): prevalence and severity, i.a.	Prevalence and severity of PRMD’s at baseline and end of program; n.s. change pre–post or between groups	− 6
Khalsa et al. (2009)	Participants summer program “Tanglewood Music Center” (Boston Symphony Orchestra’s academy), USA	15/15	15	1. Group: yoga lifestyle intervention (during 8 weeks) 2. Group: yoga and meditation only (during 8 weeks) 3. Group: no-practice-control group	RT^a^	“PRMD’s” (performance-related musculoskeletal disorders): prevalence and severity, i.a.	Prevalence and severity PRMD’s at baseline, end of program and follow-up are low; n.s. changes pre–post or between groups	− 2
Nygaard Andersen et al. (2017)	Professional orchestra musicians of Odense Symphony Orchestra, Denmark	12	11	1. Group: specific strength training (SST) 3 exercise periods á 20-min/week at the workplace for 9 weeks 2. Group: general fitness training (GFT) 3 exercise periods á 20-min/week at the workplace for 9 weeks	RT	“Pain intensity” last 7 days on VAS (100-mm), i.a.	SST group stat. sign. reduction in pain (pre 26.3 ± 22.5, post 11.4 ± 15.2 mm), n.s. reduction for GFT (pre 19.7 ± 24.0, post 13.5 ± 26.0 mm), n.s. difference between groups comparing change scores for SST and GFT	3
Sousa et al. (2015a)	Professional orchestra musicians, Portugal	39	30	1. Group: real acupoints were treated by Tuina techniques 2. Group: non-specific skin points were treated	RT	“Pain intensity” (VNRS)	1. Group VNRS pre 5.03 ± 1.87, post 0.41 ± 1.03; 2. Group VNRS pre 3.80 ± 1.80, post 3.50 ± 1.78	7
Sousa et al. (2015b)	Professional orchestra musicians, Portugal	39	30	1. Group: specific Tuina self-administered exercises (consisting of high-frequency pressure and vibration), musicians were instructed to repeat the exercises every day for 3 weeks 2. Group: sham tuina (points away from the commonly used acupuncture points)	RT	“Pain intensity” (NVS)	In group 1 (treatment) but not in group 2 (control), the pain intensity was stat. sign. reduced on days 1, 3, 5, 10, 15 and 20; difference between groups is stat. sign. on day 10 (*p* < 0.000), day 15 (*p* < 0.000) and day 20 (*p* < 0.005)	5

*CT* controlled trial, *GFT* general fitness training, *NPDIQ* Neck Pain Disability Index Questionnaire, *NVS* Numeric Verbal Scale, *n.s.* not statistically significant, *QA* quality assessment, *RT* randomized trial, *SPADI* Shoulder Pain and Disability Index, *SST* specific strength training, *stat. sign.* statistically significant, *VAS* Visual Analog Scale, *VNRS* Verbal Numeric Rating Scale, *WHQM* World and Health Questionnaire for Musicians

^a^Randomization only between the two intervention groups, not between intervention groups and control group

#### Duration of study interventions

The duration of the mostly physiotherapeutic interventions ranged from a single application given on 1 day with directly measured effects to interventions lasting 3, 5, 6, 8, 9, 12 and 15 weeks or interventions lasting up to 1 year. In one study, the treatment duration was individualized to each participant; the median treatment duration was 27 months, with a standard deviation of 16.7 months (Steinmetz et al. 2009).

#### Study population of interventional studies

Five studies explicitly included participants with complaints to investigate the effectiveness of the interventions (Sousa et al. 2015a, b; Damian and Zalpour 2011; de Greef et al. 2003; Steinmetz et al. 2009). Six studies included both musicians with complaints and musicians without complaints and investigated the prevalence and intensity of complaints before and after the interventions (Nygaard Andersen et al. 2017; Ackermann et al. 2002b; Brandfonbrener 1997; Chan et al. 2014; Khalsa and Cope 2006; Khalsa et al. 2009) or during the interventions compared to control conditions (Ackermann et al. 2002b).

#### Used outcome parameters of interventional studies

The intervention studies did not reveal evidence that the interventions were effective. No statistically significant changes in MCD were found in violinists after scapula taping, in musicians participating in yoga lifestyle interventions, or in orchestra musicians attending music didactic lectures and receiving instructions for home exercises or trigger point treatment with radial shockwave therapy combined with physical therapy or specific strength training in comparison to a control group (Ackermann et al. 2002a; Khalsa and Cope 2006; Khalsa et al. 2009; Brandfonbrener 1997; Damian and Zalpour 2011; Nygaard Andersen et al. 2017). However, two studies reported significant improvements within the treatment groups but not in the control group (Damian and Zalpour 2011; Nygaard Andersen et al. 2017). Tuina application and groningen exercise therapy did produce statistically significant reductions in pain in the treatment groups compared to the control groups (de Greef et al. 2003; Sousa et al. 2015a, b). Details are provided in Tables 4 and 5.

**Table 6:** Characteristics and results of the included studies, case studies

Author (year)	Description	Results
Anderson (1990)	Two flautists with digital neuropathy are treated with individual orthotic devices	Regression of symptoms with treatment in both cases
Belmarsh and Jardin (1996)	22-year-old male music student diagnosed with ulnar collateral ligament sprain due to overuse after changing the practice technique is treated with occupational therapy	Pain subsided since begin of treatment
Benatar (1994)	44-year-old male concert flutist with snapping at the little finger with radial subluxation of the connexus intertendineus of the metacarpophalangeal joint receives surgical approximation of the connexus intertendineus	Improvement of complaints after surgery
Demaree et al. (2017)	5 professional female upper string musicians with neurogenic thoracic outlet syndrome (TOS) underwent surgical treatment, all by first rib resection and scalenectomy, all received postoperative physical therapy: 1. 34-year-old violinist with TOS symptoms at the right side in the last 2.6 years, weekly playing time 40 h 2. 36-year-old violinist with TOS symptoms at the right side in the last 2.8 years, weekly playing time 60 h 3. 42-year-old violist with TOS symptoms at the right side in the last 3.2 years, weekly playing time 50 h 4. 37-year-old violist with TOS symptoms at the left side in the last 1.6 years, weekly playing time 50 h 5. 29-year-old violinist with TOS symptoms at the right side in the last 3.8 years, weekly playing time 50 h	All musicians were able to return to their musical career following treatment. The mean postoperative duration before they resumed their musical career was 5 months
Dommerholt (2010)	1. 19-year-old male music student (bassoon) with impairing pain in the left index finger during the bassoon playing due to a mismatch between hand anthropometry and size of the instrument receives silopad™ pressure sensitive dots 2. 26-year-old organist with persistent pain in the right wrist and thumb is treated with myofascial trigger point therapy for 4 months	1. Perfect improvement of the complaints when playing with Silopad™ 2. Approximate pain relief after 4 months
Hoppmann (1997)	19-year-old female music student (French horn) with ulnar nerve entrapment syndrome at the left elbow diagnosed due to overuse receives surgery with anterior transposition of the ulnar nerve at the elbow	Complete resolution of symptoms after surgery
Jepsen (2014)	55-year-old male contrabassoonist with radial tunnel syndrome receives physiotherapy with special emphasis on the mobilization of the radial and posterior interosseous nerves	Full improvement of symptoms after physiotherapy
Laha et al. (1978)	47-year-old male guitarist with progressive weakness and numbness of the right hand due to nerve compression of the median nerve at the elbow with a tight aponeurosis of the biceps tendon receives surgical decompression	Full improvement of symptoms after surgery
Lederman (1996)	1. Male double bassist 2. female violinist 3. male pianist 4. female music student (oboe), all with shoulder pain in connection with instrument playing with neuropathy of the thoracic longus nerve are treated with a combination of pain medication + avoidance of movements, then re-start with normal movements and muscle building	1. No improvement 2. Improvement after 10 months 3. Some improvement 4. Improvement
Levee et al. (1976)	52-year-old male musician (flute, clarinet, saxophone) with increasing tightness of muscles of the lip, mouth, throat and face is treated with 26 sessions in electromyography biofeedback	Noticeable improvement in the symptoms during the course of the treatment, no relapse during the 6-month follow-up
Manal et al. (2008)	20-year-old female music student (piano, violin) with pain in the neck and upper shoulder region and as well numbness of the thumbs is diagnosed with cervical radiculopathy and neural impairment of the thumbs and treated with complex physiotherapy and occupational therapy as well as learning correct posture on the instrument and active practice breaks in 13 treatment sessions	Noticeable improvement with regard to pain, function, mobility, i.a.
McFarland and Curl (1998)	19-year-old female musician (violin) with shoulder pain on both sides is diagnosed with rotator cuff tendonitis and treated with ibuprofen, physiotherapy, cold application, more practice breaks pauses and postural optimization	Complaints reduced from continuous to intermittent
Miliam and Basse (2009)	29-year-old male drummer with anterior tarsal tunnel syndrome receives surgical decompression	No complaints at controls after 3 months and 1 year
Molsberger and Molsberger (2012)	1. 57-year-old male pianist with pain in the right trapezius muscle and right elbow, since increased playing load is treated with playing reduction and 12 times acupuncture 2. 53-year-old female clarinetist with clinical signs of styloiditis ulnae and right humeral epicondylitis is treated with 8-times acupuncture	1. After treatment return to full playing without complaints 2. Complete improvement of symptoms after treatment
Nelson (1989)	Female violinist in her early 20 s with neck pain is treated with at least 11 Feldenkrais sessions	Improvement of complaints after 7 sessions
Nolan and Eaton (1989)	28-year-old orchestra musician (cello) with increasing playing impairing thumb pain is diagnosed with “basal joint laxity” and receives operative volar ligament reconstruction	Return to the orchestra with pain-free strength and endurance
Patrone et al. (1989)	19-year-old music student (violin) with digital nerve compression syndrome and hypermobility is treated with splint and strength exercises	2 weeks after treatment complete improvement of the symptoms. After 3 weeks, use of the splint can be reduced without the symptoms returning
Planas (1982)	Male trumpeter with rupture of the orbicularis oris receives surgical reconstruction	Full return of all functions and skills after surgery
Planas (1988)	28-year-old male music student (trumpet) with rupture of the orbicularis oris receives surgical reconstruction (and second surgery 2 months later due to residual findings)	Good function of the orbicularis oris and contentedness of the patient after surgery
Potter and Jones (1993)	Elite musician (Scottish great highland bagpipe) with numbness and tingling in the medial arm and medial hand as well as pain of right thenar eminence is diagnosed with overuse syndrome and treated with alterations in alignment of the instrument, playing technique and practice regimen	Gradual resolution of all discomfort and return to comfortable playing
Potter and Jones (1995)	22-year-old female violinist with bilateral shoulder pain during and after instrument play is diagnosed with rotator cuff tendinitis and receives treatment with rest, anti-inflammatory medication and physiotherapy	Significant improvement of the symptoms after treatment
Price and Watson (2011)	Male orchestral trombonist with postural problems and pain of the left shoulder, hand and face receives over at least 9 months a complex, not exactly defined treatment including physiotherapy, Alexander technique, osteopathy, therapy by neurologists, orthopedists, plastic surgeon, rheumatologist, orthodontist and dentist and uses special expedient (“Ergobone”)	Relapse after initial improvement of symptoms
Quarrier and Norris (2001)	1. female university trombone student with increasing discomfort, fatigue, and pain in her left hand and forearm with exceedingly small hands receives an individually made special ergonomic splint 2. 18-year-old sophomore in a music conservatory who played bass trombone with progressive pain of the left hand and relatively small hands receives initial rest treatment, physiotherapy and modification of the instrument	1. Decreased discomfort and ultimately cessation of pain 2. Disappearance of the symptoms within 3–4 weeks
Rider (1987)	34-year-old female orchestra cellist with performance anxiety and muscle fatigue is treated with a combination of music psychotherapy, biofeedback, systematic desensitization and cognitive restructuring over 8 sessions	In the follow-up reduction of the shoulder pain, improved performance attitudes, self-esteem and performance quality
Sakai (1992)	1. 17-year-old female pianist with lateral epicondylitis after increased playing of octaves receives intra-articular injection of steroids and local anesthetics, forearm tennis elbow support strap and stretching exercises 2. 22-year-old pianist with pain in the right wrist due to tendovaginitis de Quervain after repeated practice of octaves and wide-extent chords receives steroid and local anesthetic injection in the first compartment	1. Improvement by injection, not wearing the strap because of obstruction of playing, improvement by stretching exercises 2. Resolving the pain
Steinmetz et al. (2008)	44-year-old male violinist with persistent, performance-impairing left side neck and shoulder pain and extreme external rotation of violin and shoulder first unsuccessfully receives manual therapy and physiotherapy and is then treated with a multimodal pain therapy program, including manual therapy and changement of his pathological movement patterns while playing the violin, were resolved by teaching new movement patterns	Resolution of the pain, the main symptoms did not recur after several months of follow-up
Wilk et al. (2016)	34-year-old male violinist with pain, since less than 2 months and cramps in the forearm and hand muscles, receives 6 tensegrity massage sessions, 45 min each, every 3 days during 15 days plus advice: exercises stretching, improving posture habits, active forms of leisure. After the end of therapy the patient began regularly exercising at a gym	Pain on VAS decreased from 8 (of 10) before treatment to 5 (of 10) after the third treatment, to 0 after the sixth treatment and after 6 months
Wilson (1989)	24-year-old female clarinetist with symptoms in the temporomandibular joint (pain, limited excursion, lateral deviation and “catch” on the left joint upon opening), since practicing a staccato passage receives orthodontic treatment and bite splint	Pain-free clarinet playing for a period of 2 years

#### Quality assessment of interventional studies

*Pre*–*post studies without control groups* The quality assessment scores of the three studies in this category were rated between − 3 and 4 out of 15 possible points. Overall, there were significant methodological concerns. None of the studies reported sample size calculations, sufficient blinding or the use of objective, reliable and validated outcome measures. Inclusion and exclusion criteria were explicitly stated in only two studies (Ackermann et al. 2002b; Steinmetz et al. 2009). In one study, a group of participants received physiotherapy in addition to the examined intervention, but a subgroup analysis was not performed (Steinmetz et al. 2009).

*Controlled clinical trials* The quality assessment scores of the nine studies in this category ranged between − 10 and 7 out of 18 possible points. Overall, noticeable methodological concerns included not reporting on how blinding was conducted, not using reliable randomization procedures and not providing sample size calculations. Only one study (Ackermann and Adams 2003) reported an analysis or avoidance of additional treatments. Only a few studies reported an intention-to-treat-analysis or reported using all the participants in their assigned groups for the analyses (Sousa et al. 2015a, b; de Greef et al. 2003; Nygaard Andersen et al. 2017). In four studies, the outcome measurements were not considered to be objective, reliable and validated (Brandfonbrener 1997; de Greef et al. 2003; Khalsa et al. 2009; Khalsa and Cope 2006).

### Case studies

There were 28 case studies included, most of which were retrospective single case reports (Table 6). Almost all case studies described improvements in the complaints following the respective treatments or associations between risk factors and special complaints. We were unable to identify subsequent intervention studies to verify the treatment effects. The reported effects included surgical decompression procedures in nerve compression syndromes (Hoppmann 1997; Laha et al. 1978; Miliam and Basse 2009), surgical treatment in neurogenic thoracic outlet syndrome (Demaree et al. 2017), the use of individual orthotic or assistive devices (Anderson 1990; Dommerholt 2010; Price and Watson 2011; Sakai 1992; Wilson 1989), conservative combined treatments (Lederman 1996; Patrone et al. 1989) partly involving posture optimization (Manal et al. 2008; McFarland and Curl 1998; Potter and Jones 1993, 1995; Quarrier and Norris 2001) and physiotherapy with a special emphasis on mobilization of the radial and posterior interosseous nerves (Jepsen 2014), tensegrity massage and advice for positioning or leisure activities (Wilk et al. 2016). One report each exists for acupuncture (Molsberger and Molsberger 2012), EMG biofeedback (Levee et al. 1976), myofascial trigger point therapy (Dommerholt 2010) and the Feldenkrais method (Nelson 1989).

### Excluded studies

At the full-text level, 195 studies did not fulfill the inclusion criteria and were not included. Examples of the excluded studies are provided as follows. The study population included participants under 16 years of age (Vinci et al. 2015; Rodríguez-Romero et al. 2016; Stanek et al. 2017; Ioannou and Altenmüller 2015; Yasuda et al. 2016; Hagberg et al. 2005; Rodriguez-Lozano 2008; Goodman and Staz 1989; Larsson et al. 1993; Mehrparvar et al. 2012), did not state the age range of participants (Zaza and Farewell 1997; Lopez and Martinez 2013; Manchester and Park 1996; Kreutz et al. 2008; Revak 1989; Brandfonbrener 2000; Lee et al. 2012; Tubiana and Chamagne 1993; Williamon and Thompson 2006; Zetterberg et al. 1998) or provided insufficient information on the outcomes of interest when separated into age subgroups (Furuya et al. 2006; Dawson 2001). Further reasons for exclusion included that the professional status was not clearly specified (Pedrazzini et al. 2015; Takata et al. 2016; Mehrparvar et al. 2012; Brandfonbrener 2000, 2002; Tubiana and Chamagne 1993; Lederman 2003), MCDs were not reported discriminatingly (Hiner et al. 1987; Spahn et al. 2001) or complaints were not clearly separated into non-musculoskeletal regions in the reported results (Kaneko et al. 2005).

## Discussion

The study designs, terminology, and outcomes of the 109 studies included in this review were heterogeneous. The inclusion criteria were rarely mentioned throughout all study types, the analyses mostly did not check for major confounders, and the definition of exposure was often insufficient. In addition, the quality assessment of most studies included raised considerable methodological concerns. Therefore, sufficient statements cannot be provided for the prevalence, risk factors, prevention and effectiveness of treatment of MCD in professional musicians. Furthermore, a more profound differentiation, for example with regard to gender or the instrument played, is even less feasible.

In musicians´ medicine, we regularly observe that professional musicians suffer from MCD due to high physical and psychological work-related demands. The wide range in the prevalence of MCD, reported in the included studies may reflect the heterogeneous and often unclear definitions used to assess complaints in musicians. We assume that causality was already inferred due to the use of terms such as “PRMD” or by asking the study participants about complaints that were caused by or noticed while playing music. Thus, the basic assumption of causality was not questioned. In musicians´ medicine, we often lack validated assessment tools for MCD in relation to making music. However, some tools do exist, such as the Disabilities of The Arm, Shoulder and Hand (DASH) questionnaire (SooHoo et al. 2002; Hudak et al. 1996) with its optional sports/performing arts module.

From an epidemiological viewpoint, most of the included studies had only a small number of participants. Only one cross-sectional study (Fishbein et al. 1988; Middlestadt 1990; Middlestadt and Fishbein 1988) included a high number of participants (*n* = 2212). who were recruited from various US orchestras. Only this study was considered representative. However, even this study had considerable methodological shortcomings (see above).

The included studies had mostly a high risk of bias and little control of confounders. Some cross-sectional studies have been conducted on musicians in a single orchestra during a single rehearsal session. These studies likely suffered from a healthy-worker bias in addition to having little external validity or representativeness in the study results. When biographic data are gathered in cross-sectional studies, recall bias likely exists. Very few of the studies systematically assessed confounders that may have influenced MCD independently of the treatment, such as other physical loads, increased physical activity, working in the house and garden, unilateral strain by special sport discipline, carrying toddlers or providing home care to relatives. Individual preventive activities, such as sports, physical training, and relaxation, were not included as possible confounders. Additionally, correlations between instrument-specific workload (e.g., daily playing time) and complaints were not assessed. To assess the risk factors for MCD in musicians’, observational cohort studies are needed that utilize a sufficient sample size, different instrument groups, workloads, or workplaces and account for physical activity and other risk factors. Clinical trials investigating conventional and complementary medicine approaches that differentiate between instrument groups are needed to assess effective therapy options for musicians.

Comparing our results to the literature we found seven systematic reviews about MCD in adult professional musicians (Zaza 1998; Bragge et al. 2006; Wu 2007; Baadjou et al. 2016; Jacukowicz 2016; Kok et al. 2016; Vervainioti and Alexopoulos 2015). The presented review is the first attempt to investigate the frequencies, risk factors and treatment options for MCD in musicians. Zaza (1998) reported that the prevalence of PRMD in adult classical musicians was comparable to that of work-related MCDs reported for other occupations. Due to the previously mentioned lack of evidence for the prevalence of PRMD, we are unable to draw a comparable conclusion. This finding is in line with another systematic review (Bragge et al. 2006) that concluded that the evidence did not provide sufficient information regarding the prevalence and risk factors associated with PRMDs in pianists due to common methodological limitations, including sampling/measurement biases, inadequate reporting of reliability/validity of the outcome measures, a lack of operational definitions for PRMD and a lack of statistical significance testing. In her work on the occupational risk factors of MCD in musicians, Wu (2007) reported that the etiology of MCD is multifactorial in instrumental musicians; however, she also pointed out that cross-sectional studies cannot investigate causality. This differs from our conclusion, since we could not identify studies that searched for risk factors using an appropriate study design. More recent systematic reviews also found methodological concerns (Baadjou et al. 2016; Jacukowicz 2016; Kok et al. 2016; Vervainioti and Alexopoulos 2015). In contrast to our review, the authors reported results from the subjects of each study. Baadjou et al. reported that previous musculoskeletal injuries, music performance anxiety, high levels of stress and being a female who plays a stringed instrument seem to be associated with more MCD (Baadjou et al. 2016). Kok et al. found a point prevalence of MCD in professional musicians ranging between 9 and 68%, a 12-month prevalence between 41 and 93% and a lifetime prevalence between 62 and 93%. Ten out of 12 studies show a higher prevalence of MCD among women (Kok et al. 2016). However, the authors used a different scoring system to assess the quality of the included studies. In contrast to our assessment, the authors found that 13 out of 17 studies received high-quality scores (Kok et al. 2016). On the other hand, the results of the present study agree with the authors who reported that the current definition of PRMD does not provide causality of the complaints. We further emphasize the importance of using adequate and validated instruments for measuring outcomes in future studies. Jacukowicz reported on the psychosocial aspects of work, such as long hours at work, work content, high job demands, low control/influence, and a lack of social support were related to MCD (Jacukowicz 2016). Another systematic review included professional musicians as well as active members of a classical orchestra who were of at least 16 years of age. The review proposed that further research should include seven categories of stressors that affect classical instrumental musicians: public exposure, personal hazards, repertoire, competition, job context, injury/illness, and criticism (Vervainioti and Alexopoulos 2015).

## Limitations and strengths

The presented systematic review is a comprehensive attempt to evaluate the quality of the available literature on the prevalence, risk factors, and effectiveness of prevention or treatment of MCD in professional musicians. Therefore, we included published observational studies (case–control studies, cohort studies and cross-sectional studies), interventional studies (controlled clinical trials and pre–post intervention studies without control group), case reports and case series reporting clinical interventions. Because the data were very heterogeneous, an assessment of publication bias, such as a funnel plot, was not feasible. The quality assessments of the included studies were performed according to the study protocol, and if a methodological procedure was not described in the assessed article, it was recorded as not done. This was very often the case, with very few procedures being explicitly described as not having been performed. This may have led to poorer evaluations of those studies that provided less detailed reporting or publications that were older than the current reporting guidelines. The real study method might have been underestimated based on the shortcomings of reporting. The authors were not contacted about the included studies, because several of the studies were performed more than 10 years ago. Regarding quality assessment and assessment of the bias risk of the original studies, a certain allowance for interpretation and discussion is generally required. Therefore, quality evaluations were each carried out by two people, and a consensus was drawn for each study after a detailed discussion. A limitation of the present review is that to allow for a quantitative estimation, the quality assessment instruments, which were modified by the authors, and the subsequent quantitative estimation were not validated previously. The term MCD comprises a large number of very different disorders. The aim of this review was to provide an overview of this research area as well as to search for clues about the causality between music making and diseases or disorders. Any restrictions to specified clinical diagnoses would, therefore, have been purely arbitrary and, given the conceptual uncertainty of numerous primary studies with many-sided overlaps, would have represented a random and, therefore, meaningless section of a complex, interwoven area.

## Conclusions

The body of evidence regarding musculoskeletal disorders and complaints in professional musicians has grown substantially, since the publication of earlier reviews. However, studies analyzing prevalence, risk factors and effectiveness of the prevention or treatment of MCD amongst professional musicians, using today’s methodological requirements, are still missing. To evaluate the extent of associations of practice and performance burden with MCD, prospective, long-term cohort studies that properly take into account the influencing factors are still needed. To evaluate the effectiveness of specific treatment options for specific instrumental groups, prospective randomized confirmatory intervention studies are necessary. The use of well-defined diagnostic criteria for MCD in musicians is needed to avoid bias when selecting study participants. Strict and consistent diagnostic criteria would help to avoid the large variation in results. Establishing and implementing a validated and reliable set of outcome measurements is necessary. If it is possible to optimize the methodology as proposed above, relevant risk factors for MCD in musicians may be identified more precisely and allow for targeted prevention and intervention.

### Electronic supplementary material

Below is the link to the electronic supplementary material.

Supplementary material 1 (PDF 30 kb)

Supplementary material 2 (PDF 32 kb)

Supplementary material 3 (PDF 32 kb)

Supplementary material 4 (PDF 17 kb)

## Appendix 1: Search algorithms

### MEDLINE and EMBASE via OvidSP

(musician$1 OR instrumentalist$1 OR orchestra OR symphony OR music student$1 OR pianist$1 OR harpsichordist$1 OR organist$1 OR string player$1 OR violinist$1 OR brass player$1 OR cellist$1 OR bassist$1 OR violist$1 1 OR harpist$1 OR woodwind$1 OR flute player$1 OR recorder player$1 OR oboist$1 OR clarinetist$1 OR bassoonist$1 OR hornist$1 OR saxophonist$1 OR brass player$1 OR trumpet player$1 OR bugler$1 OR trombone player$1 OR tuba player$1 OR euphonium player$ OR percussionist$1 OR drummer$1)

AND

(exp musculoskeletal diseases/ OR musculoskeletal disease/ OR exp pain/ OR exp cumulative trauma disorders/ OR cumulative trauma disorder/ OR exp “Sprains and Strains”/ OR musculoskeletal OR orthopedic OR pain OR cumulative trauma disorder$1 OR overuse OR repetive strain injur$ OR PRMD)

AND

(Cross-sectional study/ OR cross-sectional studies/ OR cohort studies/ OR cohort analysis/ OR case control studies/ OR case control study/ OR observational study/ OR case reports/ OR case report/ OR intervention studies/ OR intervention study/ OR exp clinical trial/ OR randomized controlled trial/ OR systematic review/ OR risk factor/ OR risk factors/ OR therapy/ OR therapeutics/ OR exp clinical trials as topic/ OR exp “clinical trial (topic) “/ OR double-blind method/ OR double-blind procedure/ OR prevalence/ OR incidence/ OR cross-sectional stud$ OR cohort stud$ OR case-control-stud$ OR observational stud$ OR case report$1 OR intervention stud$ OR clinical trial$1 OR double-blind method OR randomized controlled trial$1 OR prevalence OR incidence OR systematic review$1 OR risk factor$1 OR treat$ OR therap$)

### CINAHL via EbscoHost

(musician* OR instrumentalist* OR orchestra OR symphony OR music student* OR pianist* OR harpsichordist* OR organist* OR string player* OR violinist* OR brass player* OR cellist* OR bassist* OR violist OR harpist* OR woodwind* OR flute player* OR recorder player OR oboist* OR clarinetist* OR bassoonist* OR hornist* OR saxophonist* OR brass player* OR trumpet player* OR bugler OR trombone player* OR tuba player* OR euphonium player* OR percussionist* OR drummer*)

AND

((MH “Pain+”) OR (MH “Musculoskeletal Diseases+”) OR (MH “Cumulative Trauma Disorders+”) OR musculoskeletal OR orthopedic OR pain OR cumulative trauma disorder* OR overuse OR repetive strain injur* OR PRMD)

AND

((MH “Cross Sectional Studies”) OR (MH “Prospective Studies+”) OR (MH “Case Control Studies+”) OR (MH “Nonexperimental Studies+”) OR (MH “Case Studies”) OR (MH “Experimental Studies+”) OR (MH “Systematic Review”) OR (MH “Risk Factors”) OR (MH “Clinical Trials+”) OR (MH “Double-Blind Studies”) OR (MH “Prevalence”) OR (MH “Incidence”)OR Cross-sectional stud* OR cross-sectional stud* OR cohort stud* OR case-control stud* OR observational stud* OR case report* OR intervention stud* OR clinical trial* OR double-blind-method OR prevalence OR incidence OR randomized controlled trial* OR systematic review* OR risk factor* OR treatment* OR therap*)

### PsycInfo + PsycArticles via EbscoHost

(musician* OR instrumentalist* OR orchestra OR symphony OR music student* OR pianist* OR harpsichordist* OR organist* OR string player* OR violinist* OR brass player* OR cellist* OR bassist* OR violist OR harpist* OR woodwind* OR flute player* OR recorder player OR oboist* OR clarinetist* OR bassoonist* OR hornist* OR saxophonist* OR brass player* OR trumpet player* OR bugler OR trombone player* OR tuba player* OR euphonium player* OR percussionist* OR drummer*)

AND

((DE “Musculoskeletal Disorders” OR DE “Bone Disorders” OR DE “Joint Disorders” OR DE “Muscular Disorders”) OR (DE “Pain” OR DE “Aphagia” OR DE “Back Pain” OR DE “Chronic Pain” OR DE “Headache” OR DE “Myofascial Pain” OR DE “Neuralgia” OR DE “Neuropathic Pain” OR DE “Somatoform Pain Disorder”) OR musculoskeletal OR orthopedic OR pain OR cumulative trauma disorder* OR overuse OR repetive strain injur* OR PRMD)

AND

((DE “Cohort Analysis”) OR (DE “Case Report”) OR (DE “Clinical Trials”) OR (DE “Risk Factors”) OR (DE “Alternative Medicine” OR DE “Medical Treatment (General)” OR DE “Physical Treatment Methods” OR DE “Relaxation Therapy”) OR (DE “Treatment”) OR (DE “Clinical Trials”)OR Cross-sectional stud* OR cross-sectional stud* OR cohort stud* OR case-control stud* OR observational stud* OR case report* OR intervention stud* OR clinical trial* OR double-blind method OR prevalence OR incidence OR randomized controlled trial* OR systematic review* OR risk factor* OR treatment* OR therap*)

### ERIC via EbscoHost

(musician* OR instrumentalist* OR orchestra OR symphony OR music student* OR pianist* OR harpsichordist* OR organist* OR string player* OR violinist* OR brass player* OR cellist* OR bassist* OR violist OR harpist* OR woodwind* OR flute player* OR recorder player OR oboist* OR clarinetist* OR bassoonist* OR hornist* OR saxophonist* OR brass player* OR trumpet player* OR bugler OR trombone player* OR tuba player* OR euphonium player* OR percussionist* OR drummer*)

AND

(musculoskeletal OR orthopedic OR pain OR cumulative trauma disorder* OR overuse OR repetive strain injur* OR PRMD)

AND

((DE “Incidence”) OR Cross-sectional stud* OR cross-sectional stud* OR cohort stud* OR case-control stud* OR observational stud* OR case report* OR intervention stud* OR clinical trial* OR double-blind method OR prevalence OR incidence OR randomized controlled trial* OR systematic review* OR risk factor* OR treatment* OR therap*)

## Appendix 2: Quality assessment tools

See online supplement.
