# Supplementary material for: Musculoskeletal disorders and complaints in professional musicians: a systematic review of prevalence, risk factors, and clinical treatment effects
Source: Int Arch Occup Environ Health. 2019 Sep 3;93(2):149–87. doi: 10.1007/s00420-019-01467-8 (PMC7007903; doi:10.1007/s00420-019-01467-8)
Supplement: Supplementary file 1 — Supplementary material 1 (PDF 30 kb) [file 420_2019_1467_MOESM1_ESM.pdf]

Quality Assessment Tool Case Control Studies

**Rater Initials and Rater Number (#1 or #2):**				
**Study identification (Author, Title, Year of Publication, Journal Title):**				
	**Criteria**	**Yes**	**No**	**Not applicable**
1.	Did the authors use an appropiate method to answer their question? (i.e., the right study design)			
2.	Was the research question or objective in this paper clearly stated?			
3.	Was the study population clearly specified and definded?			
4.	Did the authors include a sample size justification?			
5.	Were controls selected or recruited from the same or similar population that gave rise to the cases (including the same timeframe)?			
6.	Were the definitions, inclusion and exclusion criteria, algorithms or processes used to identify or select cases and controls valid, reliable, and implemented consistently across all study participants?			
7.	Were the cases clearly defined and differentiated from controls?			
8.	If less than 100 percent of eligible cases and/or controls were selected for the study, were the cases and/or controls randomly selected from those eligible?			
9.	Was there use of concurrent controls?			
10.	Were the investigators able to confirm that the exposure/risk occurred prior to the development of the condition or event that defined a participant as a case?			
11.	Were the measures of exposure/risk clearly defined, valid, reliable, and implemented consistently (includingthe same time period) across all study participants?			
12.	Were the assessors of exposure/risk blinded to the case or control status of participants?			
13.	Were key potential confounding variables measured and adjusted statistically in the analyses? If matching was used, did the investigators account for matching during study analysis?			
14.	Have confidence intervals or standard deviations/standard errors been provided?			
	**Quality Rating**			
**Total Points Rater #1:**				
**Total Points Rater #2:**				
**Total Points Consensus Decision:**				
**Additional Comments:**				
