# Supplementary material for: Musculoskeletal disorders and complaints in professional musicians: a systematic review of prevalence, risk factors, and clinical treatment effects
Source: Int Arch Occup Environ Health. 2019 Sep 3;93(2):149–87. doi: 10.1007/s00420-019-01467-8 (PMC7007903; doi:10.1007/s00420-019-01467-8)
Supplement: Supplementary file 3 — Supplementary material 3 (PDF 32 kb) [file 420_2019_1467_MOESM3_ESM.pdf]

Quality Assessment Tool Controlled Intervention Studies

**Rater Initials and Rater Number (#1 or #2):**				
**Study identification (Author, Title, Year of Publication, Journal Title):**				
	**Criteria**	**Yes**	**No**	**Not applicable**
1.	Did the authors use an appropiate method to answer their question? (i.e., the right study design)			
2.	Was the research question or objective in this paper clearly stated?			
3.	Was the study described as randomized, a randomized trial, a randomized clinical trial, or an RCT?			
4.	Was the method of randomization adequate (i.e., use of randomly generated assignment)?			
5.	Was the treatment allocation concealed (so that assignments could not be predicted)?			
6.	Were study participants and providers blinded to treatment group assignment?			
7.	Were the people assessing the outcomes blinded to the participants' group assignments?			
8.	Were the groups similar at baseline on important characteristics that could affect outcomes (e.g., demographics, risk factors, co-morbid conditions)?			
9.	Was the overall drop-out rate from the study at endpoint 20% or lower of the number allocated to treatment?			
10.	Was the differential drop-out rate (between treatment groups) at endpoint 15 percentage points or lower?			
11.	Was there high adherence to the intervention protocols for each treatment group?			
12.	Were other interventions avoided or similar in the groups (e.g., similar background treatments)?			
13.	Were outcomes assessed using valid and reliable measures, implemented consistently across all study participants?			
14.	Did the authors report that the sample size was sufficiently large to be able to detect a difference in the main outcome between groups with at least 80% power?			
15.	Were outcomes reported or subgroups analyzed prespecified (i.e., identified before analyses were conducted)?			
16.	Were all randomized participants analyzed in the group to which they were originally assigned, i.e., did they use an intention-to-treat analysis?			
17.	Have confidence intervals or standard deviations/standard errors been provided?			
18.	Was the study carried out at only one site, or if not, are results comparable for all sites?			
	**Quality Rating**			
**Total Points Rater #1:**				
**Total Points Rater #2:**				
**Total Points Consensus Decision:**				
**Additional Comments:**				

Application: Yes: Count +1 Point; No: Count -1 Point; Not apllicable: Count 0 Point; Not reportet means No
