# Supplementary material for: Musculoskeletal disorders and complaints in professional musicians: a systematic review of prevalence, risk factors, and clinical treatment effects
Source: Int Arch Occup Environ Health. 2019 Sep 3;93(2):149–87. doi: 10.1007/s00420-019-01467-8 (PMC7007903; doi:10.1007/s00420-019-01467-8)
Supplement: Supplementary file 4 — Supplementary material 4 (PDF 17 kb) [file 420_2019_1467_MOESM4_ESM.pdf]

Quality Assessment Tool Before-After (Pre-Post) Studies With No Control Group

Rater Initials and Rater Number (#1 or #2):				
Study identification (Author, Title, Year of Publication, Journal Title):				
	**Criteria**	**Yes**	**No**	**Not applicable**
1.	Did the authors use an appropiate method to answer their question? (i.e., the right study design)			
2.	Was the study question or objective clearly stated?			
3.	Were eligibility/selection criteria for the study population prespecified and clearly described?			
4.	Were the participants in the study representative of those who would be eligible for the test/service/intervention in the general or clinical population of interest?			
5.	Was the sample size sufficiently large to provide confidence in the findings?			
6.	Were study participants and providers blinded to treatment group assignment?			
7.	Was the test/service/intervention clearly described and delivered consistently across the study population?			
8.	Were the outcome measures prespecified, clearly defined, valid, reliable, and assessed consistently across all study participants?			
9.	Were the people assessing the outcomes blinded to the participants' exposures/interventions?			
10.	Was the loss to follow-up after baseline 20% or less? Were those lost to follow-up accounted for in the analysis?			
11.	Did the statistical methods examine changes in outcome measures from before to after the intervention? Were statistical tests done that provided p values for the pre-to-post changes?			
12.	Were outcome measures of interest taken multiple times before the intervention and multiple times after the intervention (i.e., did they use an interrupted time-series design)?			
13.	If the intervention was conducted at a group level (e.g., a whole hospital, a community, etc.) did the statistical analysis take into account the use of individual-level data to determine effects at the group level?			
14.	Have confidence intervals or standard deviations/standard errors been provided?			
15.	Was the study carried out at only one site, or if not, are results comparable for all sites?			
	**Quality Rating**			
**Total Points Rater #1:**				
**Total Points Rater #2:**				
**Total Points Consensus Decision:**				
**Additional Comments:**				

Application: Yes: Count +1 Point; No: Count -1 Point; Not apllicable: Count 0 Point; Not reportet means No
